# Content of attention-deficit hyperactivity disorder psychoeducation packages: scoping review

**DOI:** 10.1192/bjb.2025.10121

**Published:** 2026-04

**Authors:** David Morris, Sharifah Shameem Agha, Miriam Cooper, Kate Langley

**Affiliations:** 1 Cwm Taf Morgannwg University Health Board, Abercynon, UK; 2 Wolfson Centre for Young People’s Mental Health, Cardiff University, Cardiff, UK; 3 Division of Psychological Medicine and Clinical Neurosciences, School of Medicine, Cardiff University, Cardiff, UK; 4 School of Psychology, Cardiff University, Cardiff, UK

**Keywords:** Attention-deficit hyperactivity disorder, patient education, psychoeducation, psychosocial interventions, neurodevelopmental disorders

## Abstract

**Aims and method:**

To truly understand the efficacy of attention-deficit hyperactivity disorder (ADHD) psychoeducation, we need to know what is commonly included in it. This scoping review aims to describe the content of psychoeducation interventions for ADHD in published research. A literature search was conducted to identify relevant papers. Descriptions of psychoeducation aimed at children, parents/carers, adults and teachers were identified and compared narratively.

**Results:**

After screening, 57 papers were identified for data extraction and coding. Content themes included ‘information about ADHD’; ‘practical advice’; ‘impact of ADHD’; ‘treatment of ADHD’; ‘co-occurrence’; and ‘self-image/self-esteem’. ‘Information about ADHD’ and ‘practical advice’ were the most common themes, with variance on inclusion of other themes. Most of the identified research involved psychoeducation for parents of children with ADHD.

**Clinical implications:**

This review provides greater understanding of the content and delivery of ADHD psychoeducation. Further research could use this understanding to ascertain the efficacy of different content themes in supporting those with ADHD.

Attention-deficit hyperactivity disorder (ADHD) is a neurodevelopmental disorder with a persistent pattern of one or a combination of symptoms, such as inattention, hyperactivity and impulsivity, resulting in careless mistakes, difficulty following instructions, difficulties with organisation, being fidgety, struggling to take turns, and interrupting.^1^ These symptoms may have an impact not only on the person with ADHD, but also on their school/work performance and relationships with family members and peers.^2^ It is a common condition, estimated to affect 5.29% of the population, with a potentially chronic course progressing into adulthood (although partial remission is possible)^3^ and therefore having a significant impact on society.^4^

## The role of psychoeducation in the management of ADHD

Psychoeducation forms a key part of the management of ADHD at all ages^5^ and is a universal step in management in the UK regardless of severity of symptoms,^6^ as well as in Germany, The Netherlands, Spain and the USA.^7^

Psychoeducation is frequently delivered in combination with other psychosocial interventions, such as parent training.^8^ However, contradictory evidence suggests that psychoeducation may either dilute the effect of such interventions^9^ or make them more efficacious.^10^ The variability between psychoeducation packages is likely to influence these findings. It is therefore necessary to have a comprehensive understanding of the content of psychoeducation packages for ADHD as a starting point for understanding their benefit.

## Defining psychoeducation about ADHD

There is no consistent definition of psychosocial interventions about ADHD generally, with terms such as parent training, psychoeducation and neural feedback also lacking consistent definitions.^11^ Indeed, there is no universal definition of psychoeducation, or its modality, features or components.^12^ In a review of psychoeducation about ADHD, many studies did not attempt to define psychoeducation or demonstrate the authors’ concept of psychoeducation.^13,14^ Therefore, when analysing the efficacy of ADHD psychoeducation, previous systematic reviews have attempted to define psychoeducation as part of their inclusion criteria.^15,16^ Bäuml et al attempted a definition for the use of psychoeducation in psychosis, set goals for psychoeducation to allow patients to become ‘experts’ and relatives ‘co-therapists’, and so improving adherence, promoting relapse prevention and improving crisis management.^17^ Dahl et al utilised this definition to note that the aim of psychoeducation should be to empower patients and families to gain a sense of control over the management, contrasting with behavioural parent training, which gives the strategies to manage.^15^ Ferrin & Taylor added to this, citing also a manual for bipolar disorder psychoeducation, that a ‘good’ psychoeducation programme should be ‘evidence-based’, didactic and delivered weekly in groups, but they do not cite a research base to evidence why this is good practice.^18^ A review of the components of parent training separated definitions of parent training and psychoeducation, defining the former as providing education to parents on how to change their child’s behaviour, although it does not define psychoeducation.^9^ Other reviews that focus on efficacy of psychosocial interventions have included psychoeducation as a broad term and allowed the included papers to describe psychoeducation themselves,^10,19^ and a review that aimed to describe psychoeducation about ADHD more broadly included a descriptive review of how studies defined themselves as providing a psychoeducation intervention.^13^

In addition to describing its components, or contrasting it with other types of parent, carer or patient support, another way to describe psychoeducation is by the intended outcome. Psychoeducation was initially described as a family intervention that provided information to reduce family members’ anxiety about a relative with schizophrenia and reduce the impact of the patient’s symptoms by allowing family members to better support those symptoms experienced by the patient.^20^ Therefore, psychoeducation about ADHD can help parents better understand their child’s ADHD symptoms and therefore lessen their effect.^15^ A review of behavioural interventions for ADHD suggested psychoeducation likely has an indirect role, for example, supporting co-existing symptoms such as oppositional behaviours.^21^ Other indirect effects are as a role to inform and improve patients’ and carers’ understanding of their illness and its treatment, and to increase willingness to engage in treatment.^9–11^ However, psychoeducation is not a harm-free intervention: for example, excessive reliance on biological explanations can lead to increased feelings of stigma.^22^ Existing literature reviews on content of ADHD are limited; they suggest there is some variability in the psychoeducation content delivered for ADHD,^13,14^ although these are only briefly commented on^13^ or only available for adults with ADHD.^14^ Therefore, there is a very limited knowledge base of what information is included when delivering psychoeducation, or the effects (positive or negative) of the individual components on achieving its aims.

## NICE guidance about psychoeducation in ADHD

Guidelines for diagnosis and management of ADHD published by the UK’s National Institute for Health and Care Excellence (NICE) note that psychoeducation as part of a multimodal intervention would be of clinical benefit in individuals over 5 years of age.^6^ In the creation of its guidance, NICE conducted a systematic review of qualitative literature, which identified patient, family and professional views on the information and support needs of people with ADHD after diagnosis.^23^ That qualitative review identified the following themes:

- understanding the positives and negatives of a diagnosis
- challenges for people with ADHD
- challenges for parents of those with ADHD
- perspectives and understanding of ADHD for health professionals
- impact of ADHD in school and the teacher’s role
- experiences of services and methods of providing information and support.

However, there was no comparison with available psychoeducation packages, and the NICE evidence reviews did not state whether these themes match up with what is already included in published reports of ADHD psychoeducation packages. Additionally, NICE has recommended that further research be conducted to define the optimum length and number of sessions for efficacious parent training.^12^ The method of delivery of the psychoeducation is not specifically reflected in NICE guidance on ADHD,^6^ other than that information delivery should include parents and carers, and group parent training is recommended as per their antisocial behaviour guidelines.^24^ The European ADHD Guidelines Group’s synthesis of guidelines for adolescent ADHD found that guidance either did not specify format or stated it is left to parental preference.^7^

## Purpose of this review

To fill the gap, the purpose of the present scoping review was to describe the content of psychoeducation packages about ADHD that have been used in published research. We aimed to describe the psychoeducation delivered in terms of: (a) the modality and common themes; and (b) who the psychoeducation was aimed at: parents, teachers, or adults or children with ADHD. Our primary objective was to summarise included components of psychoeducation, identifying similarities and differences between studies and within and between multiple target audiences, as well as how psychoeducation packages overlap with the areas highlighted by the NICE guidelines. Our secondary objective was to describe the method of delivery of the psychoeducation.

## Method

### Search strategy

The PRISMA statement for scoping reviews was used to guide the study.^26^ To identify the relevant articles, Embase, MEDLINE and PsycInfo databases were searched, using the OvidSP platform. Support was provided by a Cardiff University librarian and the search was then further refined in discussion. The full search strategy can be found in Supplementary Appendix 1, available online at https://doi.org/10.1192/bjb.2025.10121. In summary, it was based on key words identified in the title, abstract or keywords to capture different terms for attention-deficit hyperactivity disorder and psychoeducation. Subject headings were identified using the OvidSP platform subject heading mapping tool. Citation search was carried out on all reviews identified by the search, and papers that were selected for review were searched for relevant citations not identified in the original search.

### Inclusion and exclusion criteria

The inclusion and exclusion criteria (detailed in Supplementary Appendix 2) were agreed between D.M. and S.S.A. before creating the search strategy. In brief, we included any research study, protocol or service evaluation published in a peer-reviewed journal in the previous 20 years (2003–2023) that investigated or described psychoeducation as the whole or a component part of an intervention or control for individuals with ADHD. We reviewed all studies that could have a psychoeducation component, including parent training programmes.

### Paper reviews

D.M. undertook review of all papers at all stages. Each paper was reviewed by another author (S.S.A. for title and abstract reviews, K.L. or M.C. for full-text reviews) and disagreements were discussed at consensus meetings. Discussions were held to reject studies where it was felt that the psychoeducation content was not described in sufficient detail or where there was duplication of material in sub-analysis or different studies were based on the same psychoeducation package. These decisions were also made in a consensus meeting between D.M., K.L. and M.C. For further information on discussion of specific programmes, see Supplementary Appendix 2.

### Data extraction and analysis

We developed a standardised form to collect data and descriptions of the psychoeducation content. Where psychoeducation was included as part of a wider mixed-methods approach (e.g. with cognitive–behavioural therapy, mindfulness, etc.) data were extracted based on the study’s own labelling of the component as psychoeducation or, where this was lacking, was judged based on criteria we derived for this study. These criteria were based on the original description of psychoeducation,^20^ recent reviews on psychoeducation^13,15^ and consensus agreements between D.M. and K.L. Included content therefore needed to meet the following rules:

- the topic should be provided in an educative, systematic manner, delivered in a didactic process as an interaction between someone with expertise in ADHD and any or all of: the patient, their carer, family and wider system, including teachers;
- the primary aim is imparting of specific knowledge to provide understanding of ADHD and empower the patient or their system in: managing the disorder and defining treatment goals and complications of the disorder.

The extracted psychoeducation components were coded in a form of thematic analysis adapted from Thomas & Harden,^26^ specifically coding each psychoeducation component to create an initial bank of codes and then adding new codes to this bank where the current codes were insufficient. The codes were then grouped into themes, with analytical themes formed organically as core concepts for the psychoeducation.

Prior to developing the set of codes, papers were grouped into the target audience for comparison. This gave five groups: (a) ‘adults with ADHD’, (b) ‘parents/carers of those with ADHD’ (in practice, this was parents/carers of children with ADHD), (c) ‘children with ADHD’, (d) ‘children with ADHD and their parents/carers’ and (e) ‘teachers’. Studies in whole or in part that spanned more than one group, for example if the target audience of the psychoeducation was teachers and carers, then that content was included in both audience groups or its components were included separately by group if delivered separately. Papers relating to psychoeducation for adults with ADHD were used to generate the first batch of codes; this was done separately by D.M. and K.L. and a consensus was reached when comparing the two coding lists. D.M. then coded the remaining papers and these were reviewed by K.L. As new codes were added, the previous papers were reviewed to see whether the new codes were relevant. Codes were chosen to summarise the psychoeducation component across the groups as succinctly yet completely as possible, and multiple codes were used where necessary, for example ‘Understanding of the ADHD diagnosis, common difficulties (including psychiatric comorbidity) and strengths’ from Hirvikoski et al^27^ was coded for ‘Diagnostic process’, ‘Presentation and symptoms’, ‘Comorbidity’ and ‘Strengths and positive elements of ADHD’. These codes were then grouped into themes.

Although the bulk of the analysis is narrative, descriptive statistics were used to help illustrate the distribution of themes in each target group and the sample as a whole. Microsoft Excel functions were used to count the frequency within the audience group that the theme occurred no more than once per paper. We used this, rather than total number of codes or themes, because some papers had more codes occurring more frequently simply because they had more detail.

Data were also extracted to provide descriptive statistics of the type of study, methodology and delivery of psychoeducation to individuals or groups. Where there was a range given for length of session, the longest time given for the sessions was recorded. Means for length of session and number of sessions were calculated only for the pure psychoeducation interventions, as there were insufficient data for when psychoeducation was a component of a multimodal intervention.

## Results

### Overview of included papers

**Fig. 1:**
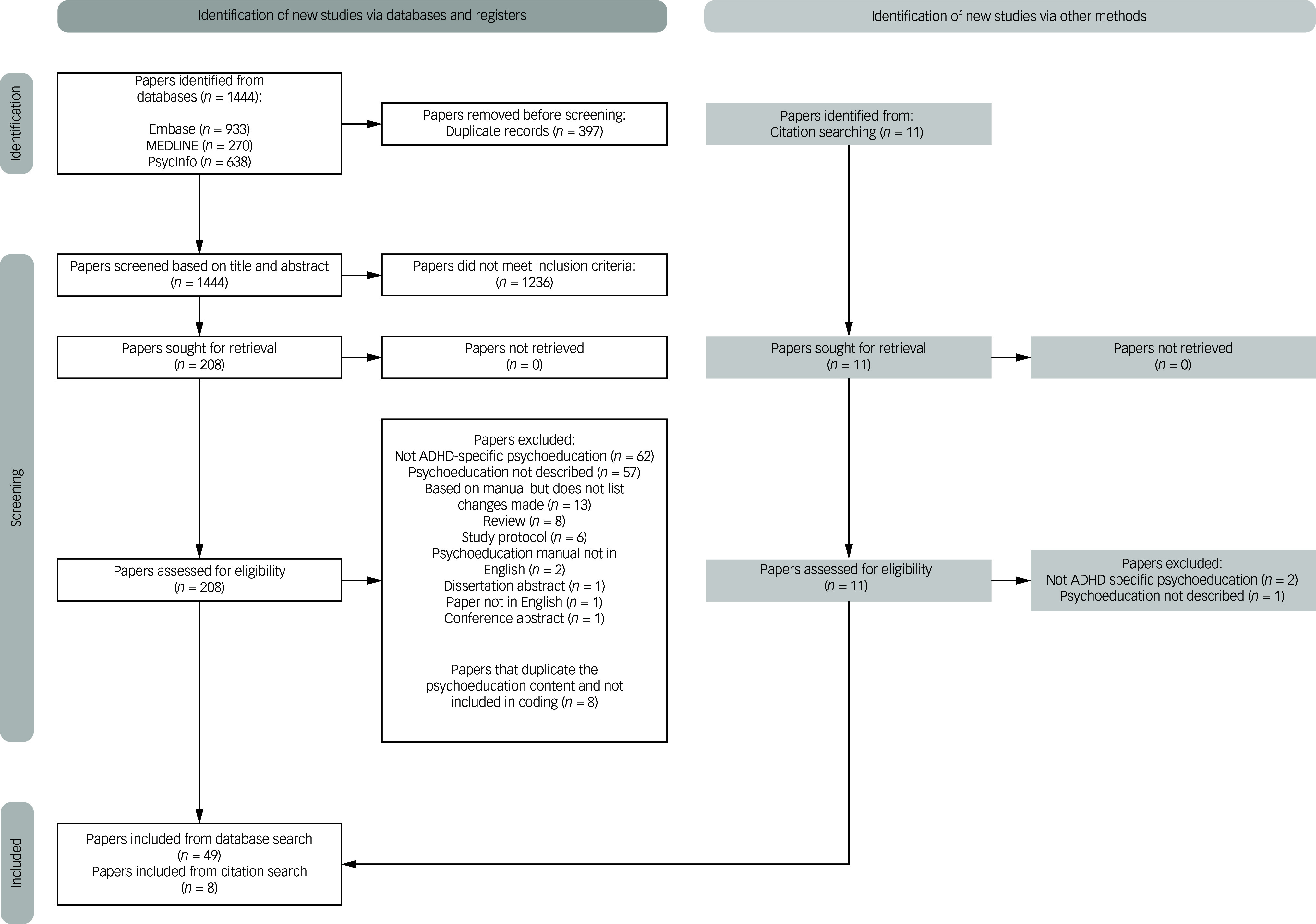
Data selection made using the PRISMA-compliant Shiny app.^29^

The titles and abstracts of 1444 papers were screened for inclusion, leaving 219 full texts that were screened for matching our inclusion criteria (11 added through citation). Following full-text screening, 65 papers (8 from citation) were included for coding and analysis (Cohen’s kappa was 0.75 at initial discussion for inclusion, with full agreement on all papers after consensus meetings) (Fig. 1). A further eight papers were removed at the data extraction stage because it had not been clear at the screening stage that the psychoeducation content was duplicated and did not add to the narrative review. The final total of 57 papers included:

- 28 RCTs
- 21 quasi-experimental pre-test/post-test studies
- 2 cohort studies,
- 2 case control studies
- 2 treatment protocols
- 1 service evaluation
- 1 non-randomised study

A full description of the included studies can be found in Supplementary Appendix 3.

The 57 papers included in the coding were broken down into the five target audience groups, as seen in Table 1.

**Table 1:** Percentage of occurrence of each theme no more than once per paper, according to target audience group

Target group (number of papers)^a^	Theme					
	Information about ADHD	Co-occurrence	Impact of ADHD	Practical advice	Treatment	Self-image/ self-esteem
Parents/carers of children with ADHD (*n* = 26)	96%	46%	50%	100%	35%	35%
Parent and child (*n* = 12)	91%	55%	64%	73%	73%	18%
Adults with ADHD (*n* = 12)	100%	60%	90%	90%	90%	50%
Teachers (*n* = 10)	91%	27%	73%	91%	64%	18%
Children with ADHD (*n* = 3)	67%	33%	100%	100%	33%	67%
All papers (*n* = 57)	96%	50%	71%	91%	59%	46%

ADHD, attention-deficit hyperactivity disorder.

a. Four papers contributed to two target groups: for example, in Hantson et al’s study,^28^ psychoeducation sessions were delivered separately to children and parents. In this review, the component delivered to children was included in the ‘Children with ADHD’ group and the component delivered to parents in the ‘Parents/carers of children with ADHD’ group.

There were 42 papers that came from North America and Europe: 13 from the USA, 7 from Sweden, 5 each from Canada and Spain, 4 each from Germany and UK, 2 from the Netherlands, 1 from Denmark and 1 from Norway. Of the remaining 15 studies, 8 came from Asia, 2 from Africa and 2 from South America (both Brazil); the remaining 3 came from Australia, New Zealand and Türkiye respectively. Studies from the Global South represented a disproportionally higher number of interventions delivered to teachers (4) and included adaptations of Western psychoeducation packages (3).

### Coding and themes

**Fig. 2:**
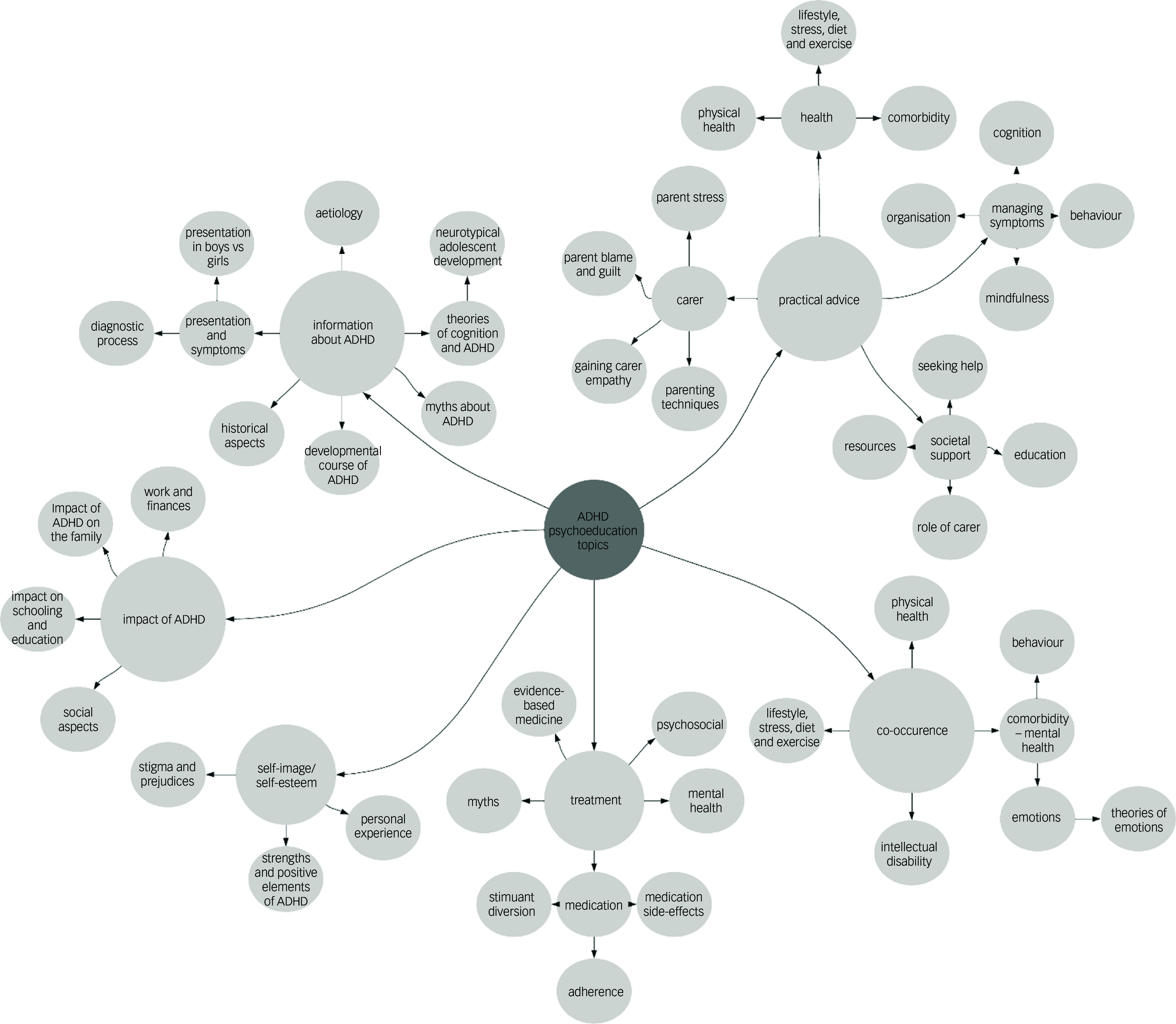
Organisation of codes into the six themes. Large circles represent the six themes; smaller circles represent the individual codes. Arrows represent the links between themes and individual codes. vs, versus; ADHD, attention-deficit hyperactivity disorder.

Using the methods described, 55 unique codes were identified. Some could be subsumed within a more general code, especially when there was more detail in the psychoeducation description being coded. For example, regarding medication, some studies simply said they covered ‘medication’ whereas others gave more detail, such as ‘Addressed concerns about addiction, long-term effects and influence on intelligence and growth’ in Bai et al.^30^ Following discussion between D.M. and K.L., six themes were derived that coalesced around all identified codes. These were: theme 1 Information about ADHD; theme 2 Practical advice; theme 3 Impact of ADHD; theme 4 Treatment; theme 5 Co-occurrence; and theme 6 Self-Image/Self-Esteem. These themes are described below and how they mapped onto the different codes can be seen in Fig. 2. The percentage of occurrence of each theme (no more than once per paper) according to the target audience group is shown in Table 1.

### Theme 1: information about ADHD

This theme represents factual information and background theory, such as causes of ADHD, proposed neurobiology, and presentation and symptoms. This theme was by far the most common (Table 1), occurring in 96% of the coded papers. The theme also includes more nuanced material directly related to or justifying the aspects of any broader intervention described by the paper, such as ‘cognitive model of ADHD’ in Vidal et al,^31^ ‘deficits in executive function may affect college students’ in Hartung et al,^32^ ‘historical aspects’ (e.g. ‘history of ADHD’ in Treacy et al^33^) and developmental course (e.g. ‘adolescent and adult outcomes and prognosis’ in Treacy et al^33^). Debunking the myths of ADHD was a further common code within this theme, and covered ‘correct any parental misconceptions’, which was present in the studies from China,^30^ Iran^35^ and Japan.^34^

### Theme 2: practical advice

‘Practical advice’ was also widely covered by the psychoeducation packages, featuring in 91% of the total coded papers (Table 1). Practical advice covered self-help advice for adults and children with ADHD, classroom management advice for teachers and parenting interventions for parents/carers of children with ADHD. A large contributor to this theme was the code ‘practical advice – managing behaviour’, featuring at least once in 22 of the 26 papers aimed at parents. One example was ‘techniques to increase positive behaviours, positive reinforcement, praise, chip economy’ in Garreta et al.^36^ Also featuring heavily was practical advice about lifestyle, stress, diet and exercise (e.g. ‘personal risk’ behaviours (internet use, exercise, sensation seeking activities, sexuality, overeating included), focus on change when needed’ in Jans et al^37^).

### Theme 3: impact of ADHD

‘Impact of ADHD’ ranged in the extent to which it featured in the studies, occurring in 71% of the total number of studies, but varying from being present in 100% of child and 90% of adult studies, but 64% of those aimed at parents/carers (Table 1). Example codes for this theme are: ‘impact on schooling and education’ (e.g. ‘academic challenges, reasons for these, and relation to social behaviour and peer’s perceptions in school’ in Mikami et al^38^); ‘social aspects’ (e.g. ‘potential and problems of people with ADHD in social life’ in Hoxhaj et al^39^); ‘impact of ADHD on the family’ (e.g. ‘situation of siblings’ in Lindstrom et al^40^); and ‘work and finances’ (e.g. ‘working life and sick leave, financial support’ in Bjork et al^41^).

### Theme 4: treatment

The inclusion of information about treatment was also widely variable, featuring in 59% of the coded papers overall, but varying from 90% of papers describing psychoeducation for adults with ADHD but only 35% of papers describing psychoeducation for parents/carers. This theme covered aspects of medication as well as psychosocial treatments. Medication-based psychoeducation also covered aspects of side-effects and debunking myths about medication. Often, the specific type of treatment was not described (e.g. ‘evidence-based treatment options’ in Risley et al^42^), which limited further interrogation. Psychosocial treatments were rarely named specifically.

### Theme 5: co-occurrence

Co-occurrence associated with ADHD was mentioned in 50% of the total coded papers. This was often broad and not clearly specified and so simply coded as co-occurrence (e.g. ‘associated problems’ in MacKay & Corkum^43^), but other papers, especially those targeted at adults or parents, were more specific. For example, the more specific code of ‘co-occurrence-mental health (emotions)’ for ‘education on depressive symptoms and treatment’ in Jans et al,^37^ or ‘co-morbidity-substance use’ and ‘co-occurrence-lifestyle, stress, diet and exercise’, with both codes being used for ‘health risk behaviours associated with ADHD (e.g., substance abuse, risky driving, sexual behaviour, overeating)’ in Schoenfelder et al.^44^ Other codes in this theme, as seen in Fig. 2, occur rarely.

### Theme 6: self-image/self-esteem

‘Self-image/self-esteem’ was the least frequently featured theme across the coded papers, included in 46% of the total. It was included in most of the studies where psychoeducation was delivered to children, featuring in 2 of the 3 papers. It was much less common (18%) in psychoeducation packages delivered to teachers and those delivered to parent and child combined (Table 1). This theme included topics such as strengths and positives of having ADHD (e.g. ‘every brain is “unique” (highlighting potential strengths and challenges)’ in Mackay & Corkum^43^) and stigma (e.g. ‘anti-stigma message whilst encouraging the teen to take ownership of ADHD-related characteristics’ in Hogue et al^81^).

## Results

### Parents/carers of children with ADHD

The largest proportion of the included studies described psychoeducation aimed at parents/carers of children with ADHD (28 papers, 49%). ‘Practical advice’ was a focus of packages for this target audience and was included in all papers, ‘information about ADHD’ in all but one; ‘treatment’, ‘co-occurrence’ (comorbidity) and ‘self-image/self-esteem’ featured in less than half of the studies. This practical advice was focused on behavioural management techniques or informing parent training, supporting education and access to resources.

### Parents and children

In the studies aimed at parents and their child (12 papers, 21%) the parents and children received the psychoeducation together. The comonest codes were ‘Information about ADHD’, ‘treatment’, whereas ‘practical advice’ was less frequent than in those aimed at just the parents, with ‘impact of ADHD’, ‘co-occurrence’ and ‘self-image/self-esteem’ featuring less frequently. There was more of a focus on items covering developmental course (e.g. ‘prognosis, development through the life span’ in Ferrin et al^46^) in packages for this group and focus on medication-based treatment.

### Adults with ADHD

Psychoeducation that was aimed primarily at those aged 18 and above with ADHD were grouped together here, and featured in 12 of the papers (21%). All themes occurred in at least 50% of these papers, with ‘information about ADHD’ featuring in all papers, ‘impact of ADHD’, ‘practical advice’ and ‘treatment’ featuring in 90%, and ‘co-occurrence’ and ‘self-image/self-esteem’ featuring in half or more. Only this cohort covered impact of ADHD on work and finances, and there was also a greater emphasis than seen in other target groups on comorbid physical health conditions (e.g. ‘lifestyle disorders and how to prevent them (sleep problems, stress, metabolic syndrome)’ in Bjork et al^41^) and psychosocial treatments, and this was usually unspecific (e.g. ‘is medicine the only way to treat ADHD?’ in Anastopoulos et al^47^).

### Teachers

The psychoeducation packages that were aimed at teachers (10 papers, 20%) were delivered to all teachers, regardless of whether or not they had a child with ADHD in their class. ‘Information about ADHD’ and ‘practical advice’ featured prominently, as did ‘impact of ADHD’, ‘treatment’, with ‘co-occurrence’ and ‘self-image/self-esteem’ featuring much less. Focus was on practical advice for managing behaviour in the classroom, (e.g. ‘teaching techniques designed to decrease inappropriate behaviours: extinction, time-out and response cost’ in Miranda et al^48^).

### Children with ADHD

The fewest identified studies were aimed at children with ADHD (*n* = 3; 5%), with ‘impact of ADHD’ and ‘practical advice’ featuring in all; Hantson et al^28^ focused on practical advice in the part of their intervention delivered only to children. ‘Information about ADHD’, specifically aetiology, also featured heavily.

### Delivery of psychoeducation

For all 65 papers (including *n* = 8 identified as duplicates) we extracted descriptive statistics on the delivery of psychoeducation, including multimodal interventions where psychoeducation was delivered alongside another intervention. Descriptive data on the specific elements of the psychoeducation were limited. In 42 studies (64.1%), psychoeducation was delivered as part of a multimodal intervention that included medication and/or other psychosocial interventions; in 10 of these studies, psychoeducation was part of ADHD-specific parent training and data describing session length and number of psychoeducation-specific sessions could not be extracted. In the remaining 23 studies (34.3%), psychoeducation only was delivered.

As regards format of delivery, 49 studies were of a multimodal group intervention with a psychoeducational component, 12 were of delivery to individuals or family groups (including 6 self-help interventions) and 1 study^48^ comprised both group and individual interventions. Within this number, only 18 delivered psychoeducation alone to a group and only these studies can be used to derive psychoeducation session length, number of sessions and size of group. Owing to the small number of studies that delivered a pure psychoeducation intervention in group format, it is not possible to create meaningful statistics for the subgroups by target audience. Across all studies, the mean length of session was 2.04 h (s.d. = 1.08), with 2 studies not reporting length of any session. The mean number of sessions was 5.11 (s.d. = 3.71), with 1 study not reporting the number of sessions delivered. Only 2 studies delivered a single psychoeducation session. The mean group size was 12.25 (s.d. = 11.38), with 8 studies not reporting the number of people in the groups. There was wide variation in group size, with larger groups for teachers and parents. In total, 12 studies described interventions delivered to individuals (parent and child dyads were not counted as individual). For individual sessions, 6 involved self-directed psychoeducation with provided information, with 4 using a self-help book or booklet (with 1 having a 2 h introductory session), 1 using a website and 1 delivered as part of internet-based cognitive–behavioural therapy. Of the 6 studies that delivered in-person sessions to individuals, the mean length of session was 0.79 h (s.d. = 0.92), with 1 study not reporting the length of sessions. There was wide variation in session length, from 5 min to 2 h in the 5 studies. The shorter sessions occurred in 2 of the 5 studies, where short episodes of psychoeducation were delivered as part of a wider consultation with a health professional regarding diagnosis and management of ADHD. Owing to the wide heterogeneity in the format of delivery, including within individual studies, we did not calculate further descriptive statistics on delivery of psychoeducation as they were not meaningful.

## Discussion

This scoping review aimed to describe the key characteristics of psychoeducation interventions for ADHD as reported in published literature accessible via major research databases. Themes on content were synthesised from 57 published papers covering a wide range of target populations, with a broad definition of psychoeducation and including interventions in which psychoeducation was part of a multimodal psychosocial intervention.

### Primary objective: to summarise included components of psychoeducation

Our main finding was that the key themes of the psychoeducation covered were: information about ADHD; practical advice on managing the disorder; co-occurring (comorbid) conditions and complications of having ADHD; treatment of ADHD; impact of having ADHD; and self-esteem and self-image. Some comparison can be made with the NICE guidelines on psychoeducation about ADHD,^6^ created through its review of qualitative research. NICE labels the intervention a ‘structured discussion’ including families and carers, rather than psychoeducation, and comments on the ambiguity of the term in accompanying documents.^12^ NICE states that this discussion could include the impacts of diagnosis, which is shared with our study’s themes of ‘impact of ADHD’ and ‘self-image/self-esteem’, and of the challenges of managing ADHD with other conditions, which is shared with our study’s theme of ‘co-occurrence’. The NICE guidance places great emphasis on the impact of symptoms, whereas this review identified that programmes being delivered place a greater emphasis on information about ADHD, i.e. what the symptoms are rather than how they affect the individual in their psychological and social life. This is especially the case for the parent and child and carers subgroup, where the ‘impact of ADHD’ theme occurred less frequently. NICE does, however, recommend that families and carers receive information on causes of ADHD, which would be included in the ‘information about ADHD’ theme. There is an emphasis in the NICE guidance on the positive and negative impacts of receiving a diagnosis, specifically mentioning issues with education, employment, social relationships and impulsivity, with comorbidities of substance misuse adding to the challenges of comorbidity and impulsivity.^6^ Although these features have something in common with the themes of ‘impact of ADHD’, ‘co-occurrence’ and ‘self-image/self-esteem’ identified in this review, they were less prominent, with the latter two themes featuring in about 50% of the included papers.

NICE emphasises the importance of modifying the environment around the person with ADHD,^6^ and this appears to be reflected in the ‘practical advice’ theme, which was present in 91% of the included papers in our review. However, NICE also specifically mentions impact on driving, which none of the included papers addressed. This is of particular note in interventions aimed at adults with ADHD, as UK driving law requires individuals to alert the authorities if they have an ADHD diagnosis or use ADHD medication.^50^ As regards ADHD medication, NICE recommends that those receiving treatment are ‘informed’ before and after their decision to take medication,^6^ and that recommendation covers topics similar to the subthemes that make up our ‘treatment of ADHD’ theme. It should be noted that much of the research included in our review was published prior to this NICE guidance and much of it is from the USA and beyond. The American Academy of Paediatric’s Clinical Practice Guideline on ADHD in children and adolescents does recommend psychoeducation, but does not provide particularly detailed guidance on what topics should be included.^51^ In summary, although we have identified that some psychoeducation packages cover those items recommended by national guidance, this is not consistent – with notable variability between the individual studies and with current psychoeducation focusing more on descriptions of ADHD and less on its impact on the individual.

The identified papers demonstrate that research into psychoeducation about ADHD is heavily parent focused, with few interventions aimed at children and young people with the disorder described in the literature. This finding is similar to that of a scoping review on psychoeducation in autism spectrum disorder.^52^ This suggests a comparative paucity of packages available to those wanting to provide research-informed age-appropriate psychoeducation for young people, despite it being an important part of the therapeutic relationship and medication regime adherence.^18,53^ Also, the identified papers were predominantly based in populations from the Global North, and those based in Asia were more about adaptations of Western psychoeducation packages for the Eastern audience. This suggests a lack of research describing psychoeducation packages specifically designed for those from non-Western backgrounds.

### Secondary objective: to describe the method of delivery of the psychoeducation

As a secondary goal of the study, we also looked at characteristics of how the sessions are delivered. The majority of psychoeducation packages were provided in group format, with an average of 2 h per session and an average of five sessions per package. This would not be purely 2 h of psychoeducation: the sense from the papers is that it includes time for an introduction to the session, a break and group discussions. There was an average of 12 people per group, although this mean has been skewed by a small number of studies with large group sizes. Removing these studies (the three with a group size over 10) would give a mean of 6.22 people per group. NICE guidance recommends more individual sessions,^6^ but the predilection for a group format may reflect efficient use of limited research resources rather than a comment on efficacy. Future studies on psychoeducation about ADHD should examine the efficacy of group versus individual delivery. Individual sessions could be briefer, for example in Lopez et al brief psychoeducation was delivered during a psychiatric clinic appointment.^54^ Again, length of session and efficacy would be a recommended area of future research.

### Strengths

This study has several strengths. It conducted a search of multiple databases and a wide array of terminology was reviewed to provide a large number of papers. It includes both pure psychoeducation interventions and interventions that delivered a clear psychoeducation component as a part of psychological therapy, mindfulness, coaching or parent training. This is especially important as our literature-informed definition of psychoeducation enabled us to include papers in which the psychoeducation component was not explicitly categorised by their authors. This allows a greater variety of information to inform our description and increases confidence in reaching data saturation. The study provides a comprehensive overview of psychoeducation content about ADHD across multiple groups, and is the first to do so for children and their parents or carers. The aims of this review, including secondary objectives, have been aligned to gaps in research identified by NICE,^6^ and will provide a baseline to inform future studies of efficacy of psychoeducation in ADHD and evaluation of services providing psychoeducation about ADHD.

### Limitations

As outlined in the aims, this scoping review is descriptive and does not comment on the efficacy of either the identified components of the psychoeducation or the mode of their delivery. To conduct a review of efficacy could be challenging: 29 of the included studies were quasi-experimental in nature, and owing to their lack of masking (blinding) and control groups it would be difficult to extract reasonable estimates of efficacy. As can be seen in Supplementary Appendix 3, there is significant heterogeneity in outcomes and outcome measures used, some focusing on ADHD symptoms and others on the impact ADHD can have on other aspects of the person and their life. This would further complicate a meta-analysis of the available data. Supplementary Appendix 3 also describes multiple weaknesses of the studies, which would further hamper the validity of any resulting statistics on efficacy, although this does not question the importance of such work.

Although our search terminology was extensive and generated a larger number of included studies than similar reviews,^13,14^ it is possible novel ADHD-specific psychoeducation has been overlooked. Owing to resource and time constraints, we were limited in our ability to review multiple databases for appropriate studies and we excluded potentially relevant conference abstracts, dissertations and grey literature, and publications from more than 20 years ago. It should also be noted that 70 studies were excluded owing to lack of detail in the psychoeducation described (either not describing the content or making undescribed changes to a manual), and it was beyond the scope of this review to be able to contact all authors for further information. Compounding this was exclusion of non-English materials, for example a small but significant number were in German.^55^ Therefore there may be elements of psychoeducation that have been missed from our descriptions and themes. This also highlights the need to include this information in the main paper or supplementary documents when publishing studies on psychoeducation.

A further limitation is due to the lack of a unified definition or standardisation of the term ‘psychoeducation’. This limits searches to identify appropriate studies. For example, additional studies were included following a citation search of Ward et al.^19^ Although their review was identified in our search, some of their included articles were not because they were described as ‘teacher training’ rather than psychoeducation. These were included following the citation searches as they matched the definition created for this study. There could be other studies that delivered psychoeducation under our definition that were not identified because the authors and publishers used broader terms not included in our search terms. A decision was made to not include extremely broad terms (e.g. training) to ensure the number of identified studies was manageable.

### Practical implications

This review might be used as a guide for those who offer psychoeducation for ADHD in commissioning a suitable package or offering their own bespoke support, by informing them of the most commonly included topics across multiple target audiences, and our comparison with NICE guideline recommendations puts this into context. Our themes can be used to create a psychoeducation syllabus that mimics the ‘current consensus’, with individual codes providing prompts for topics to cover. This is especially important with groups that have been comparatively neglected by research, such as children as the target audience. This study also serves as a foundation for further primary research and systematic reviews into psychoeducation, as outlined above. Furthermore, we note ambiguity in how psychoeducation resources were created: clearly describing this process, with end-user involvement, is important.^57^ Finally, it serves as an important reminder of the importance of reporting the content of psychoeducation programmes in research and clearly defining psychosocial modalities of treatment.

## Supporting information

Morris et al. supplementary material Morris et al. supplementary material

## Data Availability

The extracted data supporting the findings of this study are available within the article and its supplementary material.

## About the authors

**David Morris** is a child and adolescent psychiatrist at Cwm Taf Morgannwg University Health Board, Abercynon, UK. **Sharifah Shameem Agha** is a lecturer at Cwm Taf Morgannwg University Health Board, Abercynon, UK, and an honorary lecturer at the Wolfson Centre for Young People’s Mental Health, Cardiff University, UK and in the Division of Psychological Medicine and Clinical Neurosciences, School of Medicine, Cardiff University, UK. **Miriam Cooper** is a consultant child and adolescent psychiatrist, Cwm Taf Morgannwg University Health Board, Abercynon, UK, and an honorary senior research fellow in the Division of Psychological Medicine and Clinical Neurosciences, School of Medicine, Cardiff University, UK. **Kate Langley** is a senior reader in the School of Psychology, Cardiff University, UK.
