# Supplementary material for: Content of attention-deficit hyperactivity disorder psychoeducation packages: scoping review
Source: BJPsych Bull. 2025 Aug 12;50(2):163–74. doi: 10.1192/bjb.2025.10121 (PMC13150543; doi:10.1192/bjb.2025.10121)
Supplement: Morris et al. supplementary material [file S2056469425101216sup001.pdf]

Appendix 1: search strategy

1. ADHD.ti,ab,kw.
2. attention deficit*.ti,ab,kw.
3. hyperkinetic disorder.ti,ab,kw.
4. attention deficit hyperactivity disorder/
5. 1 or 2 or 3 or 4
6. patient education/
7. psychoeducat*.ti,ab,kw.
8. (patient adj2 educat*).ti,ab,kw.
9. 6 or 7 or 8
10. 5 and 9
11. 10 use medall
12. psychoeducation/ or health education/
13. ADHD.ti,ab,kw.
14. attention deficit*.ti,ab,kw.
15. hyperkinetic disorder.ti,ab,kw.
16. Attention Deficit Disorder with Hyperactivity/
17. exp Patient Education as Topic/
18. (patient* adj2 educat*).ti,ab,kw.
19. psychoeducat*.ti,ab,kw.
20. 12 or 17 or 18 or 19
21. 13 or 14 or 15 or 16
22. 20 and 21
23. 22 use oemezd
24. ADHD.tw.
25. attention deficit*.tw.
26. attention deficit disorder/ or attention deficit disorder with hyperactivity/
27. 24 or 25 or 26
28. client education/
29. parent training/
30. psychoeducat*.tw.
31. (patient* adj2 educat*).tw.
32. 28 or 29 or 30 or 31
33. 27 and 32
34. 33 use psyh
35. 11 or 23 or 34
36. limit 35 to human
37. limit 36 to last 20 years
38. remove duplicates from 37

Appendix 2: Full inclusion criteria and notes on including named ADHD multimodal psychosocial interventions.

Full inclusion and exclusion criteria

The inclusion criteria were

a) Studies describing and evaluating ADHD specific psychoeducation intervention where the psychoeducation forms the whole or part of the intervention, including as a comparator or placebo, service evaluations and descriptions of clinic protocols or development of interventions using psychoeducation.
b) b) Publication in peer-reviewed journal
c) c) Studies in English
d) d) any study design.

The exclusion criteria were:

a) published articles that were not a research study (e.g. books, book chapters, guidelines)
b) b) Studies that do not describe their psychoeducation component, or if based upon another referenced psychoeducation manual, do not specify if changes were made or do not state what changes were made, as this will not allow us to extract the syllabus used
c) c) significant amount of the psychoeducation material is not in English
d) d) Systematic reviews (though reference lists scoured for relevant studies)
e) e) studies not published in last 20 years, as it was felt this ensured studies remained relevant to current discourse.

Articles were included where they cited a referenced psychoeducation manual or material and was explicit that the original material and its delivery was not modified, or was explicit in the modifications. All papers citing parent training were reviewed, with those having non-ADHD specific interventions excluded. If the program cited was described as ADHD specific, the program was reviewed for any psychoeducation components. If there was no education around ADHD, they were excluded as being a pure parent training intervention. Where source material was unavailable or did not report sufficient information, authors were approached to provide further information.

New Forest Parenting Program

A group discussion (DM, KL and MC) regarding the inclusion of the NFPP (New Forest Parenting Program), concluded that the self-help version[57] should be included as the self-help manual was easily available and details of the psychoeducation was given. The self-help manual was reviewed, and the chapter subheadings used where they described psychoeducation. Other studies using NFPP did not provide sufficient information on the psychoeducation given to be included in either the papers or source material, although they do note psychoeducation as an important part of the programme[58].

Barkley Protocol

Many studies suggested psychoeducation based on the Barkley Protocol however, did not state adaptations made, a good example is Loren et al.[59] , which combines the Barkley[60] and another Behavioural Parenting Strategy, but did not categorically state that the psychoeducation included was that utilised in the study, in a unmodified form. Many were also based on the “defiant children” protocol such as Solan et al.,[61] and excluded as the psychoeducation was not specific to ADHD. These decisions were made in group discussion with DM, KL and MC. 4 studies were included which were based on the Barkley Protocol.

Appendix 3: Table 1. Summary of all included papers

Study	Type of study	Country of study	Participant characteristics and how recruited	Type of psychoeducation	How evaluated	Significant results	Critical appraisal
Aguiar et al. (2014) [62]	Pretest/ post test	Brazil	All teachers of 6-10 year olds in catchment area of the university were invited, 37 out of 44 agreed to take part.	1 6hour session with the entire sample, included a written manual<br><br>Psychoeducation created based on previous intervention and [60]	Knowledge test*	Significant improvement in mean scores pre and post test	Convenience sample<br><br>No evidence of impact on children with ADHD<br><br>Post test done on same day as pre-test, no longer term follow up
Anastopoulos and King, (2015) [47]	Pretest/ post test	USA	43 undergraduate students, 95% diagnosed with ADHD.<br><br>Purposive sampling from a mixture of campus based sources and word of mouth.	Group CBT and mentoring with psychoeducation components called ACCESS as part of the STAR project<br><br>90min sessions with 30 min mentor weekly for 8 sessions, followed by 2 maintenance sessions and 5-6 30minute individual sessions<br><br>Does not state size of groups	50 item ADHD knowledge* , Strategies for success measure*, ADHD cognitions test*, Cognitive response test for ADHD*, CAARS-S:L, adult version of BRIEF, BDI, BAI, Information about college performance	Significant reduction in inattentive and total ADHD symptoms (not hyperactive-impulsive symptoms)<br><br>Significant reduction in BRIEF-A scores	Quasi-experimental design<br><br>Funding sources acknowledged.<br><br>Evaluating intervention created by the authors
Au et al., (2014) [63]	RCT	Hong Kong	Recruited 17 parents of children who had been diagnosed with ADHD from a Children's Hospital and the Boys and Girl's Clubs Association of Hong Kong	Intervention was 5x2.5 hour group sessions, three telephone 20-30mn catch up sessions, and one booster session, with a group workbook. Training delivered as lectures, discussions role play, observation and DVD demonstrations.<br><br>Used a modified version of the Positive Parenting Program with modifications to "specifically target ADHD"	ECBI, PSoC, SNQ<br><br>Focus group interview	ECBI intensity subscale score significantly improved.	Small sample, with significant loss to follow up with only 6 participants. Does not state if intention to treat analysis was used
Bachmann et al., (2015) [64]	RCT	Germany	Adults not receiving medication and diagnosed with ADHD, 21 received mindfulness vs 19 psychoeducation.<br><br>Recruited from an adult adhd outpatient clinic	Psychoeducation based on a manual by [55]in German.<br><br>8 group weekly sessions lasting 2.5 hours. Doesn't state size of groups	Self and observer rated CAARS<br><br>MRI scans<br><br>Working memory task	Increased activation in right parietal lobe doing working memory exercise in mindfulness group c/w pyschoed group.<br><br>No sig difference in pre and post CAARS scales between groups but significant difference in improved scores for both groups	fMRI changes could be due to learning effect
Bai et al., (2015) [30]	RCT	China	90 parents from 89 families whose children diagnosed as having ADHD and receiving medication<br><br>Children aged 6-16 years recruited when arrived for their first hospital appointment	Comparison of 2 sessions of psychoeducation based on theory of planned behaviour and counselling and 2 group sessions of discussion with a clinician and access to an online community and a parent manual, verses general clinical counselling<br><br>Created based on theory of planned behaviour, limited description of how the program was created.<br><br>Groups lasted 40 minutes with 5-10 parents	Knowledge about ADHD*, Theory of Panned Behaviour Questions*, Medical Adherence Questions*, parent completed 4th version of ADHD-RS, likert scale of satisfaction with the program	Significant increase in condition and treatment knowledge, more positive attitude, , and more likely to seek support, and stronger intention to adhere to medication. Significantly fewer stopped medication.	No blinding<br><br>No sample size calculations<br><br>Demographic differences between groups at baseline
Bjork et al., (2020) [41]	Pretest/ post test	Sweden	48 adults with self reported ADHD and comorbid (not acute) mental illness<br><br>Recruited from clinics and	Lifestyle intervention – psychoeducation with practical exercises, including physical exercise.	ASRS, AAQoL, Lifestyle-Performance-Health (LIV) Project self report questionnaire,	Weekly physical activity improved significantly<br><br>No improvement in total quality of life score, but sub	Quasi experimental<br><br>Large attrition: 10 drop outs and 35/43 participated in the final data collection

			newspaper and radio advertisements	Groups of 6-12 lasting 90mins of 10 sessions on alternate weeks and 8 monthly follow up sessions	HADS, Maximal oxygen uptake during a submaximal cycle ergometer test	scores for Life productivity significantly increased and Relationships significantly decreased<br><br>General health significantly improved but not maintained by final measurement<br><br>Significant decrease in depressive symptoms	Evaluating intervention created by the authors<br><br>Limited blinding, those who led the groups also performed the tests
Christiansen et al., (2014) [65]	RCT	Germany	58 children randomised to self management or neurofeedback. In both groups parents and children received psychoeduation, however only parent psychoeducation is described and children psychoeducation references a manual in German.<br><br>Recruited from a university outpatient department of psychology	5x 100min sessions in groups – size of groups not stated<br><br>Psychoeducation based on [66]	German version of 3rd edition CPRS and CTRS, PSI, PS, CASSS,<br><br>Start, stop and doses of medication<br><br>Qb-tes (computer based test of ADHD symptoms)<br><br>Children's test battery of attention assessment<br><br>Self concept interview<br><br>KINDL-R quality of life scale<br><br>Perceived criticism scale*<br><br>Quantitative EEGs	Outcome measures do not describe effect of psychoeducation as all members receive it.<br><br>CPRS and CTRS subdomains significantly improved. No reports on remaining measures in this study.	Preliminary results and therefore limited reporting on the results of the study.<br><br>No blinding of participants or therapists<br><br>Full results of study not published and study status listed as unknown on www.clinicaltrials.gov
Ciessielski et al., (2020) [67]	Pretest/ post test	USA	304 parents with 159 children aged 6-12 with a parent reported diagnosis ADHD without co-existing neurodevelopmental disorder<br><br>Recruited from hospital clinic providing Behavioural Parent Training (BPT)	Eight session manualised BPT group, Based on Barkley[60], Loren et al. [59] and Wells et al. [68] Does not state group size or length of session	HSQ, DBSI	Significantly reduction on all aspects of HSQ except task performance<br><br>Reduced parental stress led to improved non familial transactions and task performance on DBSI	57% had complete data<br><br>Not assessed impact on child symptoms<br><br>Limited demographics collected and hard to tell if representative sample.
Corkum et al., (2019) [69]<br>†	RCT	Canada	58 teachers-students dyads (students with ADHD aged6-12) recruited from 7 English speaking schools were randomised into teacher training (28) and waitlist control (30).<br><br>Children were on a stable dose of medication or no medication.	Online 6 week course (new session each week) with combination of videos, presentations, supporting documents and a discussion board with an ADHD coach<br><br>Group size of 25-19	Treatment tracking form*<br><br>3rd editions of CTRS and CPRS, IRS, TSR, PSR<br><br>Acceptability questionnaire*	Significant decrease in CTRS post intervention and 3 month follow up<br>No significant<br><br>High level of acceptability and satisfaction.	Between group difference in that teacher waitlist group were older by on average 5.3 years<br><br>Some medication changes in children through the study<br><br>Limited blinding and no objective measures
Daley and O'Brien, (2013) [57]	RCT	UK	64 parents with 43 children aged 4-11undergoing assessment for ADHD. Allocated to self help intervention or delayed self help intervention.	Self help materials "step by step help for children with ADHD" (Daley et al. (2011)[70] based on NFPP with a 2h small group induction + weekly reminder	PACS, ADHD-RS, Direct child observation, Parental GHQ12, PSoC, Fidelity index*	Significant improvement in PACS, ADHD-RS, and PSOC scores	7 lost to follow up<br><br>Small scale study<br><br>Coauthor of paper is co-author of the manual

			Recruited from a clinic where children were referred for ADHD assessment and not yet medicated.				
Daley et al., (2021) [71] † Uses the same treatment manual as Daley and O'Brien (2013)[57] and so not included in coding	RCT	UK	52 children aged 6-10 years old recruited from 11 CAMHs clinics who had received medication and diagnosed with ADHD Received the same self help book as above	Self help materials "step by step help for [70]children with ADHD" with supportive phone call and DVD + weekly reminder and TAU vs TAU alone	PSoC, GHQ-12, ECBI, SNAP-IV, FSI, Speech samples to test for high expressed emotion, SAMBA, VADPRS, child and parent form of CHIP,	Significant improvement in parenting efficacy, FSI (on completers only), reduction in negative comments parents made about their child, teacher reports of child relationships with their peers,	More loss to follow up in treatment arm High loss to follow up throughout, and especially at long term follow up (20/52) No power calculation No Bonferroni adjustments
Danforth (2006) [72]	Treatment protocol	USA	No participants Tailored to manage the specific problems faced with ADHD and ODD based on previous research. Psychoeducation based on Barkley [60]	Psychoeducation on ADHD, ODD coercion and social learning principles prior to introducing parent training and use of a flow chart to manage behaviour	Nil	Nil	No studies evaluating this training
Dopfner et al., (2004) [73]	RCT with adaptive elements	Denmark	75 children aged 6-10 referred to a University outpatient clinic diagnosed with ADHD randomly assigned to medication+ psychoeducation and then behaviour therapy alone or combination, or behaviour therapy+ psychoeducation if no contraindication for the assigned group. For those receiving behavioural therapy only but ineffective, received medication and psychoeducation	All received 6-8 weeks of psychoeducation lasting 45-60 minutes. Does not cite resources for the psychoeducation or state how it was created.	Parent and teacher FBB-ADHD, German versions of the CBCL, TRF, HSQ, HPC, parent and teacher IPC,	Parent FBB-ADHD, CBCL attention, externalizing, internalizing and total subscales, HSQ and HPC, and teacher rater FBB-ADHD, TRF attention, externalizing, internalising and total scores were significantly reduced for all. All initially started on medication showed significant decreases in parent and teacher FBB-ADHD, CBCL, IPC, HSQ and HPC	Doesn't state how psychoeducation was developed Only 6-8 weeks at each treatment stage, which is brief and not reflect real world treatment decisions Use of low dose medication (15mg MPH)
Ferrin et al., (2014) [74]	RCT	Spain	44 received psychoeducation vs 37 received blinded placebo of a support group of 8-10 families over 12 weekly 90min sessions. Recruited from children attending a CAMHs clinic diagnosed with ADHD	8-10 parent groups of 12 week 90minute sessions described as a psychoeducation program Adapted from a psychoeducation package for bipolar disorder and literature describing psychoeducation	Spanish versions of the CPRS-S, CTRS-R:S, SDQ, CGI, short form of PSI, EQ-5D, PedsQL	Significant improvement in CPRS and CPRS inattention/ cognition subdomain at 12 weeks, with subdomain improvement only at 12 months. SDQ prosocial subdomain significant at 12 months. Significant improvement with CGI over time.	No independent rating of ADHD symptoms
Ferrin et al., (2020) [46]	RCT	UK	69 families with children aged 5-18 diagnosed with ADHD 35 diagnosed ADHD received psychoeducation vs 34 diagnosed ADHD control – both allowed treatment as usual including medication. Recruited consecutively from a CAMHs clinic	Groups of 7-10 families of six sessions lasting 2 hours Described as a psychoeducational program Developed from previous psychoeducation for schizophrenia and bipolar disorder, and used in Ferrin et al(2014) [74], though there are some differences	CPRS-R:S, CTRS-R:S, SDQ, PSI, CGI, CGAS	In treatment group over time showed significant improvement for total symptoms on CPRS and subdomains except oppositional or psychopathology subdomain.	Small sizes, primary outcome was not blinded – and cites previous studies showing reduction in effect when blinded. Not clear what TAU was provided. No Bonferroni corrections applied

Fields and Hale, (2011) [75]	Case/ control –	USA	Recruited from a single urban primary care centre of children aged 7-12 years diagnosed with ADHD. 20 children and their parents received psychoeducation vs 20 on waiting list	5x60 minute group sessions weekly with parents and children receiving intervention at same time but separately<br><br>Does not state how psychoeducation package was developed.	VADPRS, VATPRS	Significant improvement in parent rated but not teacher rated scale	No consistent T2 point – 1-3 months<br><br>Selection bias<br><br>Small sample<br><br>Unclear how many people recruited, how if the 20 were children and parents or just the children<br><br>No evidence base for psychoeducation<br><br>Selection bias – study was non randomised and family received intervention if willing
Fogler et al., (2020) [76]	Service evaluation	USA	Adaptation during a pilot trial to telepsychology due to COVID-19<br><br>20 children aged 5-11 recruited from two tertiary children's hospitals following initial diagnosis of ADHD.	5-8 families in each group in 4 sessions doesn't state length of sessions<br><br>Program reported in Nissley-Tsiopinis(2023) [77] which integrates motivation interviewing into traditional BPT	Content fidelity checklist<br><br>Satisfaction questionnaire<br><br>Qualitative interviews	Highly satisfied<br><br>Themes of qualitative interviews: not needing to travel or find childcare, concern about disrupted flow of conversation due to mute/unmute, inhibited about sharing personal information via telepsychology, concern about interrupting others.<br><br>Fidelity no different to in person scores.	Small sample from privileged backgrounds, with 13/20 families completing the satisfaction ratings<br><br>Findings specific to the pandemic era.
Foubister et al., (2020) [78]	Pretest/ post test	UK	146 Parents of children aged 6-12 diagnosed with ADHD<br><br>Recruited from all those referred to Parents in Control program on diagnosis	6x 2 hour sessions with groups of up to 10 parents, with final review session after 6 week break.<br><br>Uses Parents in Control parenting training program which includes psychoeducation, developed locally	ECBI, PDH	Significant reduction in ECBI and PDH scores	Risk of selection bias as only 37% of those invited participated<br><br>No objective measures
Garreta et al., (2018) [36]	Pretest/ post test	Spain	Parents of 21 children, from a single hospital environment, children aged 6-12 diagnosed with ADHD<br><br>Recruited from a single clinic of all those referred with possible ADHD symptoms.	Parental training with psychoeducation component with children prescribed methylphenidate commenced two to four weeks before the program<br><br>Intervention consisted of 10 sessions lasting 90minutes of 4 to 9 families. No reference for the parent training program	CBCL, PS	With exception of defiant disorder and somatic disorders, all aspects of CBCL significantly improved. Significant decrease in all aspects of the PS.	No objective measurements.<br><br>22% drop out<br><br>No funding/ declarations<br><br>No reference or comment on how the program was developed or by whom.
Gummus et al., (2020) [79]	RCT	Turkie	172 parents randomised by family into parent psychoeducation group and TAU.<br><br>Recruited from people who attended a child and adolescent psychiatric clinic	43 coupled parents in two psychoeducation sessions of 45-60minutes with 15 minutes of questions and 5 minutes of warm up<br><br>No description of how psychoeducation was developed	CaSS	Significant reduction in CaSS scores in treatment group compared with TAU group at 6 months	Control group had treatment as usual, not documented what this was.<br><br>CaSS reliability score <0.7, so uncertainty of reliability of this measure<br><br>Ages of children not documented
Hantson et al., (2012) [28]	Case control study	Canada	33 children with ADHD attending daily summer camp vs 15 children with ADHD who could not attend. Camp	Parents of children attending summer camp had individual psychoeducation, four x 2hr sessions. Does not state size of groups.	Parent completed WFIRS, CGI, IPR	Significant improvement in all aspects except WFIRS risk activity index	Risk of selection bias, Controls selected because parents could not drive them to the camp or had prior engagements

			focuses on social skills, music therapy, art therapy, play therapy.<br><br>Recruited from a University linked ADHD clinic	Does not state how psychoeducation was developed or informing literature.			
Hartung et al., (2022) [32]	Pretest/ post test	USA	30 undergraduate and post graduate students with self-rated ADHD symptoms, 27 of whom had a previous diagnosis.<br><br>Purposive sampling through flyers listing the project and by referrals from an on campus clinics	Group CBT study skills intervention<br><br>6 group and 3 individual sessions. 2 pure psychoeducation sessions over 6 weeks. Not stated group size or length of sessions.	DSM-5 current checklist, self-report, WFIRS, CAARS-S:L, Organisational, Time Management and Planning (OTMP) self report*	DSM-5 Current Checklist Inattention scores significantly improved<br><br>CAARS-S:L total ADHD scores significantly reduced<br><br>WFIRS showed significant decrease in impairment<br><br>OTMP score significantly improved	Quasi-experimental design<br><br>No objective measures, all self reported<br><br>Funding sources reported and no conflicts declared.<br><br>Evaluating intervention created by the authors
Haugan et al., (2022) [49]	RCT	Norway	100 adolescents aged 14-18 year olds with ADHD or subthreshold ADHD randomised to CBT group therapy vs medication only TAU. Both received psychoeducation prior to randomisation.<br><br>Recruited from CAMHs out patient clinics linked to a university.	All received 3x1 hour sessions for parent and child and then one day session for parents and teachers<br><br>CBT based intervention group then 12 sessions lasting 90 minutes with 4-6 per group<br><br>Parent and child psychoeducation based on NICE guidance, not stated how parent and teacher psychoeducation was informed	Self, parent and teacher CGAS and CGI-S, IVth edition of ADHD-RS, BRIEF, self and parent report of WFIRs, BRIEF, SCARED, SMFQ, GPES, RSES, ASWS	No significant findings	Some purposive sampling to achieve numbers, including those with 'behaviour problems' rather than ADHD<br><br>Content of psychoeducation not standardised across the treatment participants.<br><br>Poor adherence to CBT group, with 43% attending all sessions
Hirvikoski et al., (2015) [27]	Pretest/ post test	Sweden	108 adults, 51 with ADHD and 57 significant others. Coded with adult group<br><br>Recruited from adult psychiatric clinics	Group psychoeducation using CBT principles called PEGASUS<br><br>8 group session of 20-30 individuals, lasting 2.5hours with written information	Feasibility questionnaire, ADHD 20 questions (knowledge quiz), QAFM, BDI, BAI, PSS, RSES, AAQoL, BAS	Significant improvement in ADHD knowledge Significant decrease in BAS	Evaluating intervention created by the authors<br><br>Psychoeducation not standardised across groups, not attending all lectures.<br><br>Loss to follow up
Hirvikoski et al., (2017) [80]<br><br>Not included in final coding as psychoeducati on more fully described in Hirvikoski et al., (2015) [27]	RCT	Germany	97 people, 48 with ADHD and 49 significant others vs TAU of 89: 39 with ADHD and 43 significant others<br><br>Recruited from specialist neurodevelopmental clinics	PEGASUS psychoeducation as per Hirvikoski et al.,, (2015)[27]	Evaluation questionnaire, ADHD 20 questions (knowledge quiz), SWLS, HADS, QAFM, BAS	Significant increase in knowledge vs increase in TAU group but not sustained in ADHD group on follow up.<br>HADS improved in significant others.	Evaluating intervention created by most of the authors<br><br>Small sample size<br><br>3 month follow up results for secondary measures not reported, a large proportion of participants lost to follow up
Hogue et al., (2021) [81]<br><br>Utilises MIP from Hogue et al., (2014)[82] and Hogue et al., (2016)[83] however this study also uses the CASH-AA intervention as in Hogue et al., (2016)[84] and so this was the one included in coding.	RCT	USA	Cluster randomisation: therapist randomised to deliver Changing Academic Support in the Home for Adolescents with ADHD (CASH-AA) and Medication Integration Protocol (MIP) verses CASH-AA alone in 2:1 ratio.<br><br>145 children aged 12-18 meeting diagnostic criteria for ADHD from two community clinics, one hospital clinic, one community substance use clinic	MIP: Individual families. Doesn't state length of sessions. Some psychoeducation used to help families make decisions around medication<br><br>CASH-AA is a family based psychosocial intervention to help with academic settings, and includes a psychoeducation component.<br><br>Both described as flexible without fixed number of sessions.	CASI-A, ADHD diagnostic scale of the MINI, CBCL, SRDS, School grades and self report of grade point average, HPC, Medication use	CASH-AA + MIP improved inattentive symptoms and delinquent acts more overall, and more attendance in non substance users. In substance users CASH-AA only showed greater declines in caregiver report hyperactive symptoms and externalising.	56% of families initially invited didn't complete the study (mostly due to not meeting ADHD screening eligibility).<br><br>Some authors involved in the creation of either or both interventions in this study.

			and one dual substance use and mental health clinic				
(Hogue, et al., 2016) [83]is a pilot evaluation of the MIP and not included in coding due to duplication†	Cohort with historical control	USA	26 recruited, of which 14 took part were recruited from High schools and community-based agencies who referred young people with conduct problems. The historical controls were recruited from the same streams for a previous study	MIP tasks delivered as 5 minutes each per session of 10 sessions, however its use is intended to be flexible.	CASI-A, MINI, and Psychiatric Service logs	MIP significantly more likely than HC to complete the psychiatric evaluation, be prescribed any medication and be prescribed ADHD medication.	MIP cases more likely to have been referred from a clinic, and therefore treatment seeking. Significant variability in fidelity of intervention.
Hoxhaj et al., (2008) [39]	RCT	Netherlands	96 children aged 4-12 recruited from outpatient mental health clinic and diagnosed by a psychiatrist. If parents preferred BPT or behavioural problems persisted after medication, referred to the study.<br><br>Randomised to BPT+routine clinical care (48) vs TAU only (48)	Manualised behaviour parent training based on (Barkley, 2006(and [86]<br><br>12x120 minute sessions with 6 parents in a group.	5 parent identified target behaviours and severity rated<br><br>CBCL, CPRS:RS, PSI	Target behaviours and CBCL externalising and internalising subscale showed significant improvement in BPT+TAU group. Significant improvement in both groups for CPRS:RS	16.7% of families declined, perceiving unable to fit BPT into schedule. Another 11.1% refused to participate in groups.<br><br>If one parent (usually father) refused then family did not participate in study.<br><br>No objective measurement of symptoms
Van den Hoofdakker et al., (2007) [85]	RCT	Germany	81 medication free adults with ADHD. 40 received psychoeducation with zen relaxation exercise vs 41 with mindfulness.	Psychoeducation modified from the manual by [55]in German.<br><br>8 weekly sessions for 2.5 hours. Does not state group size.	CAARS observer and self rated, BSI, BDI, SF-36, FFMQ	Significant improvement in FFMQ scores in mindfulness group.<br><br>Both groups improved significant in in attention scale in pre/post test analysis	Author of psychoeducation manual used also part of this study<br><br>Randomisation and blinding not described<br><br>17 lost to follow up.
In de Braek et al., (2017) [87]	RCT	Netherlands	Adults diagnosed with ADHD, 12 received goal management training plus psychoeducation vs 15 received psychoeducation only<br><br>Referred from an outpatient clinic	2 hours group sessions and 1 individual session for 12 weeks. Groups of 6-8 people.<br><br>Does not state how psychoeducation topics were decided and no references included.	CFQ, SCL-90, Zoo map from the behavioural assessment of the dysexecutive syndrome, blinded clinician using CIBIS/C	Clinician rated scores for cognition improved.<br>.	Small sample sizes<br><br>Control and intervention group were similar, but psychoeducation group half an hour shorter, with an additional final session
Jans et al., (2015) [37]	RCT	Germany	144 mothers and their child (children aged 6-12) where both mother and child have ADHD.<br><br>Mothers treated with group psychotherapy plus individual psychotherapy vs supportive counselling. Then all received parent-child training which had no psychoeducation components<br><br>Recruited from 5 university departments of child psychiatry	Group psychotherapy included psychoeducation with DBT principles. 120minute sessions weekly to monthly with a total of 22 sessions. 6-9 mothers in each group<br><br>Used an adult ADHD psychotherapy manual in German (Hesslinger et al 2004), psychoeducation directed at mothers to manage own ADHD<br><br>Children received a therapy program as reported in [73] and so this aspect not included in coding	K-SADS-PL - ADHD and ODD subsections, HSQ, SDQ, Maternal observer rated CAARS, SCL-90	No difference between groups for children's ADHD or wider symptoms	control (supportive psychotherapy which has some unspecified psychoeducation component driven by maternal request) is an active treatment but not superior to the multimodal treatment.<br><br>No objective measure of children's ADHD<br><br>Poor compliance with any of the treatment modalities

Jones and Chronis-Tuscano (2008) [88]	RCT	USA	Teachers from six schools were block randomised to receive intervention or waiting list.<br><br>Schools recruited from phone lists	Group sessions (group size, length of session or frequency not stated).<br><br>Included handouts on ADHD and daily report cards with small group work on constructing report cards for hypothetical students<br><br>Psychoeducation created by an advanced doctoral student with experience with ADHD	Teacher knowledge about ADHD – 25 item true/ false questionnaire*<br><br>Teacher use of classroom behaviour management strategies<br><br>Intervention satisfaction	ADHD knowledge significantly increased in treatment group<br><br>No change in reported use of classroom behaviour techniques.	No objective measure of use of strategies – might hide improved use due to responder bias.<br><br>No measure of impact on children with ADHD<br><br>For both groups knowledge of ADHD improved<br><br>Some differences in baseline characteristics (more male and special education teachers in waitlist group_
De Jongh et al., (2019) [89]	Development and evaluation of a training program	South Africa	65 teachers for 5-6 year old children from 57 schools in an urban and semi-rural district	Teachers received two half day or 1 full day workshops.<br><br>Psychoeducation developed based on a literature review of similar programs for teachers of 5-6 year olds.	Qualitative feedback	Positive feedback received, and able to use the manual for teachers to use for others including other teachers and parents. Helped with misconceptions and awareness of how ADHD was managed in multiple settings.	No objective measure of effectiveness.
Kousha and Karodi (2019) [35]	Pretest/ post test with control group	Iran	60 mothers of 8-12 year old children diagnosed with ADHD and on a therapeutic dose of methylphenidate, 21 received intervention and 24 received waiting list control<br><br>Unclear how mothers were recruited.	Positive Parenting Program group, described as an educational program, with 10-15 parents for 8 two hour sessions.	PEDS-QL	Significant improvement between pre and post test PEDS-QL scores in intervention, but not sustained at 3 months due to improvement in control group	No monitoring of ADHD medication use in trial, which could explain improvement seen<br><br>No measure of ADHD severity or impact on symptoms<br><br>Small sample sizes with 14/60 lost to follow up
Lantz et al., (2021) [45]	Pre test/post test	Sweden	125 families consisting of 113 children aged 5-12 diagnosed with ADHD and 125 parents<br><br>Recruited from CAMHS outpatient clinics	2x 1.5 hour sessions in groups of 12 children and 12 parents.<br><br>Based on the SKILLS psychoeducational program used in Meyer et al. [90] but adapted for younger children and parents.	CSQ-8, parent version of ASRS-A, CSDS, attitude to diagnosis and medical and psychological treatment*, qualitative questionnaire	Better rated by children than parents, who had a 50/50 split on whether useful or not, or met needs. Majority would recommend the intervention.<br><br>Majority felt the program had helped children to deal with their problems<br><br>Significant improvement in parent attitudes to diagnosis	No objective data<br><br>No data on between group differences on use of medication
Lasisi et al., (2017) [91]†	RCT	Nigeria	84 primary school teachers given an 75 teachers in control group on a waitlist.<br><br>Teachers recruited from public and private schools. Meet sample size calculations.	Uses WHO MhGAP-IG module on behavioural disorders. initial 3hr training with a booster session of 1.5hr 2 weeks later.<br><br>Delivered using presentations, clinical vignettes, role plays, small group discussions and videos.	SRAQ, KBIQ,	Significant difference in post intervention scores between the two groups on SRAQ and KBIQ	Teachers in intervention group significantly older, had more years of teaching experience and smaller class sizes, control group more likely to have recognised ADHD in a child, and taught more with ADHD. 1 extra private primary school in recruitment for control group.
Latouche and Gascoigne (2019) [92]	Cohort vs wait list control	Australia	274 primary school teachers across 10 schools. Those available in June 2016 received intervention, those available next month were waitlist control<br><br>Not stated how chose the 10 schools	2hr 15 minute training session with 15 minute break, PowerPoint delivered.<br><br>Half session psychoeducation, half session of classroom management strategies Based on published manual of classroom management strategies.	KADDS, TSES	Significant improvement in all scores, with some drop off (not significant) after post test,	No randomisation<br><br>Follow up varied from 30-52 days<br><br>Waitlist group was 3.7 years younger, and may have selection bias as allocated on availability to receive intervention

			out of the 300 locally available.	No documentation on where other aspects of psychoeducation were sourced.			
Lindstrom et al., (2022) [40]	Pretest/ post test	Sweden	585 parents of children aged 3-17 with ADHD recruited, pre and post interventions measurements and results stratified by parents ADHD symptom severity	5 x 3 hour lectures of groups of 25-35 parents<br><br>Recruited from an already running psychoeducation program based on Svanborg, Thernlund, Gustafsson, Hägglöf, Poole, *et al.*, 2009). This current study includes an extra 3 hour session	Knowledge quiz<br><br>SDQ, PSS, Parental attributions*	Parents ADHD symptomatology did not impact on any outcome (other than odds of incomplete study data)	Few ADHD symptoms reported,. And lower than expected for population<br><br>Limited impact of ADHD on parental functioning overall
Lopez et al., (2005) [54]	Pretest/ post test	USA	90 children aged 6-17 and their families, with a diagnosis of ADHD or depressive disorder or both, assessed four monthly for at least four months<br><br>Psychoeducation modelled on an adult version on this treatment algorithm developed by one of the authors, and used information from a variety of sources.	Enrolment in an individualised program with the child's carer determining content,<br><br>Psychoeducation given at clinical contact, and includes information provided by psychiatrist or clinical assistant, each contact lasted on average 16.5 minutes, with a total of 106.5 minutes on average during the study.	Percentage receiving each material<br><br>Parent satisfaction questionnaire<br><br>Child/adolescent satisfaction questionnaire	Only descriptive statistics used.	No measurement of efficacy.<br><br>51% and 41% return of questionnaires<br><br>No consistency in who received what psychoeducation, although the aim was to provide tailored psychoeducation.<br><br>Intervention created by one of the authors.
MacKay and Corkum (2006)[43] †	Pretest/ post test	Canada	25 children aged 8-12 diagnosed with ADHD, 22 of which prescribed stimulants<br><br>All recruited from a single ADHD clinic	Groups of 5 in a 2 hour workshop using multiple mediums described as a psychoeducation/demystification workshop	Children's knowledge and opinions questionnaire*<br><br>Parent's perspective questionnaire*	All aspects of the children's knowledge and opinions questionnaire significantly improved except opinion of alternative treatments.	No control group, cannot tell if children would have gained this knowledge through their own research or treatment as usual.<br><br>No descriptive statistics of the 4 recruited but didn't participate (due to scheduling conflicts)<br><br>Limited information about how the workshop was created.
McCarty et al., (2015) [93]	Protocol with a subanalysis from an ongoing RCT	USA	Children (aged 5.5-12.9) stratified by younger (5.5-9.9 and 10-12.9 years) and their caregivers with moderate ADHD severity, received telemental health care vs control group of a single telepsychiatry consultation with recommendation<br><br>111 received intervention of vs 112 control<br><br>Recruited from primary care providers in rural areas.	Intervention consisted of psychiatry input through video teleconferencing, and local community therapists providing caregiver behaviour training. Psychiatrists decide on a treatment algorithm pathway which determines what the local caregiver behaviour training provides. Includes 6 sessions of psychoeducation.<br><br>Psychoeducation developed from a psychopharmacological textbook[94]	Quiz scores and attendance<br><br>Descriptive statistics of the interventions<br><br>Client Satisfaction questionnaire	Caregivers attended 5.3 of the 6 medication and psychoeducation sessions and 5.1 of the 6 caregiver behavioural training sessions.<br><br>Adherence to protocol 91.6+/-9.5% reliability<br><br>Satisfaction with the treatment was 38/40<br><br>Further results in van der Stoep et al.[95]	Results demonstrating outcomes presented at conference<br><br>Unable to separate effects of each component<br><br>Lead author also created the intervention
Meyer, et al., (2022) [90]	RCT	Sweden	93 adolescents (15-18) received group skills training based on DBT and CBT called the Structured Skills Training Group vs 91 receiving manualised group psychoeducation called SKILLS<br><br>Multi centre RCT over 7 sites recruiting adolescents aged 15-	Followed SKILLS intervention consisting of three 2 hour sessions. Does not state group size	ASRS-A, CSDS, Impact of ADHD Symptoms*, GQL, FFMQ, SDQ, HADS, PAS, KSQ, Treatment Acceptability questionnaire*	Only pretest/post-test Structured Skills Training Group experienced reduction in ADHD symptoms and functional impairment for self and parental reports, and parental only in the SKILLS group. Pretest/post test improvement in sleep for SKILLS no	Evaluating intervention and control created by the authors<br><br>No adherence assessment of control group<br><br>Poor adherence in active group<br><br>Control group had more sessions<br><br>Large drop out

			18 with a diagnosis of ADHD			between group difference.	No blinding
Meyer, Zetterqvist, et al., (2022)[96]<br><br>Subanalysis of Meyer and Ramklint et al., (2022) [90] and therefore not included in coding	RCT sub analysis	Sweden	Sub analysis of follow up data of 71 receiving DBT training vs 57 receiving SKILLs psychoeducation control, as per Meyer and Ramklint et al., (2022) [90]	As per Meyer and Ramklint et al., (2022) [90]	As per Meyer and Ramklint et al., (2022) [90] But also including the emotional dysregulation question from the Impact of ADHD Symptoms* and CSDS	Treatment condition more effective for those with higher severity of hyperactivity or conduct problems or emotional dysregulation	Only one question on emotional dysregulation rather than a wider scale<br><br>No assessment of confounders including socioeconomic status and parent factors.
Mikami et al., (2020) [38]†	RCT	Canada	172 families of children aged 6-11 in primary school with ADHD and social problems. 84 parents received Parental Friendship Coaching –vs Coping with ADHD through Relationships and Education (CARE)<br><br>Recruited from schools, clinics and hospitals.	10x90minute sessions with 6-7 in each group<br><br>Parental friendship coaching contains no specific ADHD psychoeducation<br><br>CARE is psychoeducation plus space for parents to share individual approaches, in this study adapted to address friendship issues	Short version of FQQ, QPQ, SSIS, Observed friendship task in lab	PFC had significantly more pro social behaviours on QPQ and teacher SSIS. PFC more pro social on parent SSIS at 8 months	High median income families with higher median education<br><br>PFC developed by the lead author. Those who developed CARE not part of the study.
Smit et al., (2022)[97]<br><br>Sub analysis of Mikami et al., (2020) [38], and so not included in coding	RCT	Canada	As per Mikami et al., (2020) [38]	As per Mikami et al., (2020) [38]	Coding of parent and child behaviours in observed settings<br><br>CBCL	No statistically significant findings	Small sample size<br><br>Few fathers in sample<br><br>Lab observations may not represent real world situations.
Miller and Brooker (2017) [98]	Pretest/ post test	Canada	34 Parents and teachers of children with ADHD aged 5-14, does not state number of children<br><br>Recruited from advertisements in local business and parenting publications and local ADHD services.	8 weekly sessions up to 2 hours of mixed mindfulness and psychoeducation with a 5.5 hour silent retreat, in groups of approximately 11 people<br><br>Based on Mindfulness Based Stress Reduction [99] with ADHD psychoeducation which isn't described on how created	PHQ-9, GAD-7, PSS, FFMQ	Significant reduced GAD-7, and non-reactivity to inner experience, observing and describing subdomains of FFMQ. PSS described as significantly reduced but p=0.07	Updates to psychoeducation materials with each cycle of the group, lack of consistency in intervention.<br><br>Only one instructor for the course<br><br>Lack of confirmation of ADHD diagnosis<br><br>No long term follow up<br><br>Doesn't state where information for psychoeducation is sourced.
Miranda et al., (2006) [48]	Cohort study	Spain	Children referred by paediatrician with potential ADHD, diagnosed and included if met study criteria.<br>17 children identified to match the age (8-9), sex and academic level to receive medication, to 17 who received a psycho-pedagogical intervention, and 16 control.<br><br>Those receiving medication recruited from a university children's neurology department. Others recruited from a training program for teachers.	Teachers of the children diagnosed with ADHD were given 8x 3hour sessions over four months.<br><br>Psychoeducation used was from a previous study by the lead author	Abbreviated CTRS, IOWA CTRS, SPI, CSI	Significant improvement in Inattention-Disorganisation and Hyperactivity-Impulsivity subscale of CSI in teachers over time vs control, no difference between the intervention groups, and in both CTRS, and in SPI medication vs control.	No randomisation<br><br>Small groups with between group demographic differences<br><br>Small doses of methylphenidate used.<br><br>Different recruitment sites could mean that there are demographic differences not recorded.<br><br>Evaluating author's own psychoeducation program.

Monastra (2005) [100]	Pretest/ post test	USA	In 1st aspect of study identified that parental fears,, discomfort with brevity of the assessment process, development of adverse side effects, lack of information about ADHD. This informed 2nd part of study, recruited 658 families with children (aged 6-20) who had ADHD and declined or discontinued medical treatment in past three months<br><br>Recruited from a single clinic	Parents provided with psychoeducation manual "Parenting Children With ADHD: 10 Lessons That Medicine Cannot Teach" by [101]<br>Decisions to initiate medication linked to the neurobiological assessments fed back to parents.	~Medication adherence at 6 weeks, 6 months, 12 months and 24 months	70% commenced medication, and 95% of those remained on medication 2 years after	Evaluating intervention and psychoeducation manual created by the author<br><br>Limited statistics with no significance calculations
Montoya et al., (2014) [102]	Cluster randomised non blinded study	Spain	Parents of 208 children aqed 6-12 year old diagnosed with ADHD and randomised into medication + parental psychoeducation or medication alone<br><br>Based on Barklay (2002) and adapted for the study.<br><br>Recruited from clinics that did not have a psychoeducation intervention	Groups of 5-6 parents of four weekly sessions with 1 final session after 5 weeks. Each session was 90minute including standardised slides, manual with parental reading material<br><br>Described as parental psychoeducation	Monitoring of persistence with pharmacothera py<br><br>Parent version of ADHD-RS-IV, CGI-ADHD-S, Parent version of WFIRS	No significant difference to time to withdrawal of medication<br><br>Statistically significant improvement in parent ADHD-RS-IV and its inattention sub score, greater satisfaction with pharmacologic intervention,	No baseline demographic statistical comparisons. Younger, more girls, more single parents, more anxious, less ODD less learning disorders in control group<br><br>Study terminated early and underpowered due to difficulty in recruitment, didn't reach required sample size.<br><br>Authors receive funds from, and editorial support from drug companies that sell ADHD medication.
Nagae et al., (2019) [103]	pre-post test with TAU control group, non randomised	Japan	15 families in group psychoeducation vs 24 families who received TAU<br><br>Does not say how recrutied	Parents and children in groups of 5 families in 5 90 minute sessions which included a lecture, discussion, recreation and homework, with focus on discussion between families on experiences with medication<br><br>Psychoeducation tool created by the authors	Child and Parent versions of SAMBA, CAQ, 4th edition of ADHD-RS, APGAR, CSQ-8	Significant changes were child SAMBA resistance to medication decreased, CAQ scores increased, child APGAR decreased,<br><br>Parent reported SAMBA benefits of medication score increased.	No statistical comparison between control group and intervention group, just description of statistics<br><br>Non randomised, people consented to which arm of the trial (this could be reflection of real life scenarios)<br><br>Psychoeducation limited to medication management.<br><br>No analysis of adherence to medication<br><br>Testing intervention created by the authors
De Oliveira et al., (2018) [104]	Pretest/ post test	Brazil	25 health professionals who work with ADHD, 35 adults treated for ADHD and 181 others<br><br>Recruited via email lists and social media	Information booklet on ADHD, based on the main author's previous research and professional critique of the original booklet	Knowledge assessment questionnaire designed for study.	Significant increase in knowledge scores	Evaluating intervention created by the authors<br><br>No longer term follow up
Padilla and Parsons (2019)[105]<br><br>Uses the same treatment manual as Daley And O'Brien (2013)[57] and so not included in coding	Pretest/ post test	USA	8 families with 8children aged 9-15 from two outpatient clinics who were diagnosed with ADHD but not yet enrolled in treatment as usual	Used the same manual as Daley and O'Brien (2013) [57] but also received weekly phone calls and 2hr orientation session and guided the reading	Parent SNAP-IV	Only descriptive statistics used and no calculation of significance	Limited statistics used and very small sample size

Pettersson et al., (2017) [106]	RCT	Sweden	Adults diagnosed with ADHD and stable on medication (if prescribed), assigned to Internet CBT in a self help format (13), Internet CBT with weekly group therapy sessions (14) or a waiting list controls (18)<br><br>Recruited from psychiatric clinics or those referred to a neuropsychological clinic	Commercially available CBT package called In Focus, made up of 9 modules which includes an informational component	CSS, BDI, BAI, AIM-A, COPM	Significant difference with CSS between iCBT self help and waiting list, but not between iCBTgroup and waiting list.<br><br>No between group differences 6 months follow up. Posttreatment and follow-up scores maintained in iCBT-self help group.	Didn't meet sample required for power calculation by 9<br><br>CBT packaged developed in collaboration with main recruiting site and where the authors are based.<br><br>Patients in all three treatment groups had medication changes
Risley et al., (2020) [42]	Pre test/ post test	USA	132 parents of 3-6 year old children attending BPT groups offered through a hospital children's clinic. Analysis of data collected for the clinic.	Program was a variant of that by Loren et al.,[107]<br><br>Sessions had 8-12 families receiving 8 sessions, doesn't state how long they lasted.	IRS, HSQ, VADPRS Parental confidence to manage target behaviour	Significant improvements in all results after completion of BPT	No objective measures<br><br>Outcome measures collected on final group session, no prolonged follow up.
Ryan et al., (2015) [108]	Pretest/ post test	UK	172 parents whose children aged 4-18 with diagnosed or suspected ADHD were recruited and directed to a website<br><br>Recruited from outpatient CAMHs clinics	Psychoeducational website created by a company that manufactures ADHD medication	Descriptive statistics pre and after 1 month	Majority of carers accessed the website once or twice.<br><br>65% cited lack of time to access if not accessed<br><br>74% browsing for general information<br><br>Parents using the website showed increased knowledge	Don't know how long access website, other sources of information.<br><br>Recall bias, those returning to study likely remembered to access website<br><br>Doesn't state how website was developed, and risk of bias towards medication based treatments if created by medication manufacturing business.<br><br>40.5% of sample didn't access the website
Sarraf et al., (2011) [109]†	RCT	Iran	33 teachers were randomised into a workshop group, and 34 did not attend the workshop but received booklets instead Recruited from local primary schools.	Two day workshop, each session 5 hours long.<br><br>Does not state how workshop was created other than by the authors, but booklet created by delphi method	ADHD knowledge and attitudes*	Significant increase in attitudes and function between the group	Does not describe randomisation methodology<br>Those receiving the booklet were more likely to have more years of education, and more knowledge of ADHD and knowledge of function.<br>No measured impact on young people with ADHD<br>Evaluated intervention created by the authors
Schoenfelder et al., (2020) [44]	Pretest/ post test	USA	15 children (8 on medication) aged 13-17 and their family recruited from primary care centres and hospital outpatient clinics via flyers and community mental health practices	Curriculum developed through multidisciplinary collaboration between clinical psychologists, physicians from outpatient psychiatry, adolescent medicine and neurodevelopmental clinics at a large children's hospital.<br><br>Interactive large group lecture following a quiz	ADHD stigma questionnaire, ADHD knowledge questionnaire*, Treatment intent*, Domain concern*, ADHD treatment attitudes questionnaire*, CSQ-8, Open ended feedback and a Focus group	Post test significant increase in ADHD knowledge for parents and teens, and Parent acceptance of medication, reduced willingness to use and diets and cognitive games.	Quasi-experimental design<br><br>Small sample size and cannot account for variances in demographics.
Shah et al., (2019) [110]	Pretest/ post test	India	11 families of 7-14 year olds diagnosed with ADHD<br><br>No information on how people were recruited	Group training for 10 sessions, lasting 90minutes held weekly via zoom<br><br>Used group parent training created for this study which included psychoeducation	Qualitative interviews<br><br>VADPRS	Qualitative feedback on convenience, and group interaction. Improved awareness of ADHD, reduction in guilt associated with causation of	Small sample size of 8 children, and 30% drop out rate<br><br>1 family described as an 'outlier' and excluded from analysis

						ADHD, reduction in blaming the child, improved parent child relationship, improved communication, improved self competency, able to convey things more clearly to the teachers, reduction in stress, able to learn from experiences of others.<br><br>Significant improvement of all VADPRS sub scores except conduct and anxiety or depression	Evaluating intervention created the authors
Shimabukuro et al., (2017) [34]	Pilot study – one cohort with qualitative feedback	Japan	5 Japanese mothers and one grandmother recruited through a support group for mothers with parenting difficulties	All mothers met with the lead researcher for 2 hours per week for 8 weeks<br><br>Psychoeducation was NFPP with additional psychoeducation on ADHD, stress management and coping skills for parents, based on Treacy et al.,[111]	Participant feedback	More support with stress management and cognitive restructuring, more psychoeducation<br><br>Acceptable to use group format limited to mothers only	
	Pre test/ post rest		19 mothers with children aged 6-13 with ADHD or above concern on SNAP. 2 children prescribed medication<br><br>Recruited through advertisements in local newspapers, local clinics, and a public talk on ADHD	11 week group lasting 2 hours with 4-7 mothers per group.	Japanese versions of SNAP-IV, CBCL, GHQ-30, PSI, PS, PLOC	Significant decrease in SNAP-IV, aggressive behaviour subscale of CBCL, child domain and total stress on PSI, PS scores improved	No objective measures<br><br>Small sample
Svanborg, Thernland, Gustafsson, Häggöf, Poole et al., (2009) [112]	Rct	Sweden	99 children aged 7-15 years old received atomoxetine or placebo. In both groups parents received psychoeducation.<br><br>recruited from outpatient clinics	4 x3hr groups sessions for the patient's care givers/ Does not state frequency of sessions or group size. Includes lectures, group discussions, problem solving, modelling and role plays<br><br>Psychoeducation created by an uncited professional education manager. Formed basis for treatment manual as [39]	Version IV of ADHD-RS, CGI-S, CGI-I	Significant improvement in atomoxetine vs placebo on ADHD-RS and CGI-I.	Parents wanting immediate symptom relief were excluded.<br><br>No description of how the psychoeducation package was designed.
Svanborg, Thernland, Gustafsson, Häggöf, Schacht et al., (2009) [113]<br><br>Subanalysis of Svanborg, Thernland, Gustafsson, Häggöf, Poole et al., (2009) [112] and so not included in coding	RCT subanalysis	Sweden	As above – sub analysis of [112]	As above – sub analysis of [112]	Achievement domain of the Parent form of the child edition of CHIP, FSI, ASCR, FTF, I think I am scale of self-esteem, CDRS-R	Statistically significant improvement in the achievement domain, FSI in both groups (though less in the placebo group), small change in ASCR and larger decrease in FTF scores.	Smaller samples size than needed to detect meaningful difference on CHIP-CE

Tarver et al., (2022) [114]<br><br>Sub-analysis of Daley (2021) [71] and so not included in coding	Qualitative study	UK	As per Daley (2021)[71]	As per Daley (2021) [71]	Interview schedule based on initial interview	Six themes:<br>Parental desire to learn more<br>Acquisition of new skills<br>Flexibility of the intervention<br>Self help vs traditional therapist led intervention<br>Barriers to engagement in the home environment<br>Earlier access to help	Interviewer was someone involved in the study, could give a response bias.
Treacy et al., (2005) [111]	RCT. Stratified to single or two parent families	New Zealand	63 parents from 42 families of children aged 6-15with ADHD assigned to treatment of Parent Stress Management groups or wait list control.<br>Recruited from a paediatric ADHD research clinic	Group parent stress management program with session on ADHD education. Group size of 8-14 parents, meeting 9 weekly sessions lasting 2 hours per session<br><br>Doesn't state or reference how the manual was developed	PSI, BDI, PS, PLOC, short form of FAD, LWMAT, BSSQ, Consumer satisfaction questionnaire*	Significant reduction in PSI, PS and BDI, mostly seen in mothers and only verbosity subscale of PS in fatehrs	No correction for multiple tests<br><br>Almost 53% loss to follow up at 12 months
Vidal et al., (2013) [31]	RCT	Spain	Adults diagnosed with ADHD receiving medication and one family member but had ongoing symptoms: 17 received group psychoeducation vs 15 in group CBT<br><br>recruited from an ADHD clinic	12 weekly sessions lasting 2 hours in groups of 7-8 with 1 session with 1 family member<br><br>Psychoeducation program not referenced or state how topics were decided	ADHD-RS, CAARS-S, CGI-S self report and clinician version, BDI, State subscale of the STAI, QLESQ	All measures improved significantly, including symptoms over time, no between group differences.	Small group size with some differences: more men, more divorced, more combined in CBT group though not significant<br><br>Evaluating CBT intervention created by one of the authors
Zheng et al., (2020) [115]	RCT	China	211 children aged 6-11 diagnosed with ADHD via screening. 116 assigned an intervention group and 95 control. Intervention group consisted of systematic training with psychoeducation for parent and teacher<br><br>Recruited via a screening questionnaire for all pupils at the recruited schools.	Parent training: 4 weekly 2 hr sessions,<br><br>Teacher training: 4 2hr weekly sessions<br><br>Doesn't state group sizes<br><br>Psychoeducation based on a simplified version of Family School Success	MARS, Chinese version of SNAP-IV	MARS showed significant adherence and SNAP-IV scores significant improved	Randomisation based on school, no blinding<br><br>No description of control

Table 1 abbreviations:

Abbreviations:

* created for the study

† found through citation search

AAQoL = Adult ADHD Quality of Life Scale, ADHD-RS = ADHD Rating Scale, AIMA = ADHD Impact module -Adult, APGAR = Family Appearance, Pulse, Grimace, Activity, Respiration health assessment, ASCR = Appraisal of Stress in Child-Rearing, ASRS = Adult ADHD self report scale, ASRS-A = Adult ADHD self-report scale for adolescents, ASWS = Adolescent Sleep-Wake Scale, BAI = Beck's Anxiety Inventory, BAS = Burden Assessment Scale, BDI = Beck's Depression Inventory, BPT = Behavioural Parent Training, BSI = Brief Symptom Inventory, BSSQ = Brief Social Support Questionnaire, BRIEF = Behaviour Rating Inventory of Executive Function, CAARS = Conner's Adult ADHD Rating Scale, CAARS-S = Conner's Adults ADHD Rating Scale Self-Report: Short Version, CAARS-S:L = Conner's Adult ADHD Rating Scale Self-Report: Long Version, CAQ = Child Adherence Questionnaire, CAMHs = Child and Adolescent Mental Health, CASI-A = Comprehensive addiction severity index for adolescents, CASSS = Child and Adolescent Social Support scale, CBCL = Achenbach Child Behaviour Checklist, CDRS-R = Children's Depression Rating Scale-Revised, CGAS = Children's Global Assessment Scale, CHIP = Child Health and Illness Profile, CIBIS/C = Clinician's Interview-Based Impression of Severity and Change, CFQ = Cognitive Failures Questionnaire, CGI-ADHD-S = Clinical Global Impression-ADHD Severity score, CGI-I – Clinical Global Impression – Improvement, CGI-S = Clinical Global Impression Scale, COPM = Canadian Occupational Performance Measure, CPRS = Conner's Parent Rating scale, CPRS-R:S = Conner's Parent Rating Scale Revised, CTRS = Conner's Teacher Rating Scale, CTRS-R:S = Conner's Teacher Rating Scale Revised Short Version, CSS =

Current Symptoms Scale – Self Report-form, CaSS = Caregiver Stress Scale, CSDS = Child Sheehan Disability Scale, CSI = Child Symptoms Inventory, CSQ = Caregiver Strain Questionnaire, CSQ-8 = Client Satisfaction Questionnaire, DBSI = Disruptive behaviour stress inventory, ECBI = Eyberg Child Behaviour Inventory, EQ-5D = EuroQoL five dimension, FAD = Family Assessment Device, FBB-ADHD = Fremdbeurteilungsbogen für Auf-merksamkeitsdefizit-Hyperaktivitätsstörung, FES – Family Empowerment Scale, FFMQ = five Facet Mindfulness Questionnaire, FSI = Family Strain Index, FTF = Five to Fifteen questionnaire, FQQ = Friendship quality questionnaire, GAD-7 = Generalised Anxiety Disorder-7, GPSES = General Perceived Self-Efficacy Scale, GQL = Global Quality of Life Scale, GHQ-12 = General Health Questionnaire 12, GHQ-30 = General Health Questionnaire 30, HADS = Hospital Anxiety and Depression Scale, HPC = Home Problems Checklist, HSQ = Home situations Questionnaire , IPC = Individual Problem Checklist, IRS = Impairment Rating Scale, IPR = Index of Peer Relations, KADDS = Knowledge of Attention Deficit Disorders Scale, KBIQ = Knowledge of Behavioural Interventions Questionnaire, K-SADS-PL = Kiddie- Schedule for Affective Disorders and Schizophrenia-Present and Lifetime Version, KSQ = Karolinska Sleep Questionnaire, LWMAT = Locke-Wallace marital adjustment test, MARS = Medication Adherence Report Scale, MINI = Mini International Neuropsychiatric Interview, NFPP = New Forest Parenting Program for ADHD, PAS = Pressure Activation Stress Scale, PACS = Parental account of childhood symptoms, PEDS-QL = Paediatric Quality of Life Inventory, PDH = Parenting Daily Hassles, PHQ-9 = Patient Health Questionnaire-9, PLOC = Parental locus of control scale, PS = Parenting Scale, PSS = Perceived Stress Scale, PSI = Parenting Stress Index, PSoC = Parental Sense of Competence, PSQ = Connors parent symptom questionnaire, PSR = Parent Satisfaction Rating, QAFM = Questions About Family Members, QLESQ = Quality of life enjoyment and satisfaction questionnaire, QPQ = Quality of Play Questionnaire, RSES = Rosenberg's Self Esteem Scale, SAMBA = Southampton Medication Behaviour and Attitudes Scale, SCARED = Screen for Child Anxiety Related Emotional Disorders, SCL-89 = Symptom Checklist-89, SDQ = Strengths and Difficulties Questionnaire, SF-36 = 36-Item Short Form Survey, SMFQ = Mood and Feelings Questionnaire – Short Version, SPI = School Problems Inventory, SNAP-IV = Swanson, Nolan and Pelham Version IV Scale Score, SNQ = Service Needs Questionnaire, SRAQ = Self-report ADHD Questionnaire, SRDS = National Youth Survey Self-Report Delinquency Scale, SSIS = Social Skills Improvement System, STAI = State/Trait Anxiety Inventory, SWLS = Satisfaction with life scale, TAU = Treatment as Usual, TRF = Achenbach Teacher Report Form, TRS = Connors teacher rating scale, TSES = Teachers' Sense of Self-Efficacy, TSR = Teacher Satisfaction Ratings, VADPRS = Vanderbilt ADHD Parent rating scale, VADTRS = Vanderbilt ADHD teacher rating scale, WFIRS = Weiss functional Impairment Rating Scale

Appendix 4: Tables of results, coding of papers groups by target audience

Table 3: Parents/carers of children with ADHD

Study	Psychoeducation descriptions	Code 1	Code 2	Code 3
Au et al., (2014) [63]	Causes	Aetiology		
	Risks and protective factors	Aetiology		
	Parental Role	Practical advice - role of carer		
	Effective treatment Options	Treatment		
Bai et al., (2015) [30]	Manifestation of ADHD	Presentation and symptoms		
	Diagnostic process of ADHD	Diagnostic process		
	Definition of disorder and corrected misunderstanding of disease	Presentation and symptoms	Myths of ADHD	
	Potential causation/ risk factors	Aetiology		
	Impact on children's lives that necessitates intervention/ negative effects on the life course	Impact of ADHD	Developmental course of ADHD	
	Pharmacological and non pharmacological treatments	Treatment - medication	Treatment - psychosocial	
	Pharmacological mechanisms, therapeutic effects, effectiveness and side effects	Treatment - medication		
	Barriers to adherence	Treatment - adherence		
	Addressed concerns about addiction, long-term effects and influence on intelligence and growth	Treatment - medication	Treatment - medication side effects	
	Importance of persistence in medication and when to stop medication	Treatment - medication		
	General parenting skills and behaviour management strategies, including changing the environment and lifestyle, skills for managing ADHD symptoms and positive discipline	Practical advice - behaviour	Practical advice - lifestyle, stress, diet and exercise	Practical advice - parenting techniques
	Improving diet and sleep	Practical advice - lifestyle, stress, diet and exercise		
Christiansen et al., (2014) [65]	Information on ADHD	Presentation and symptoms		
	Handling problem behaviour	Practical advice - behaviour		
	Token economies	Practical advice - parenting techniques		
	Joint parent-child play	Practical advice - relationships		
	Timeout and 1-2-3 magic	Practical advice - behaviour	Practical advice - parenting techniques	
	Handling problem behaviour in public	Practical advice - behaviour		
Ciesielski et al., (2020) [67]	Diagnosis of ADHD	Presentation and symptoms	Diagnostic process	
	Neurobiology and executive function deficits	Aetiology	Theories of cognition and ADHD	
	Behavioural difficulties associated with the diagnosis	Co-morbidity - mental health (behaviour)		
	Evidence based treatment options	Treatment		
	Principles of social learning theory and behaviour modification, ABCs and reinforcement	Practical advice - parenting techniques		
	Use of praise, ignoring and special playtime	Practical advice - parenting techniques		
	Giving effective commands	Practical advice - parenting techniques		

	Creating a contingency management system	Practical advice - parenting techniques		
	Homework strategies	Practical advice - education		
	Daily report cards	Practical advice - education		
	Effectively using time out and response cost	Practical advice - parenting techniques		
	Managing behaviours in public places	Practical advice - behaviour		
Daley and O'Brien (2013) [57]	What are the symptoms of ADHD?	Presentation and symptoms		
	Further information about ADHD (recommended reading)	Practical advice - resources		
	Theories about why children have ADHD: genetics and the brain	Aetiology		
	Behaviour strategies	Practical advice - behaviour		
	Children with ADHD have strengths too	Strengths and positive elements of ADHD		
	Children with ADHD who are also temperamentally sensitive	co-morbidity - mental health (emotions)		
	Other ideas to help children with ADHD who lost their temper	Practical advice - co-morbidity		
	Staying calm	Practical advice - parenting techniques		
Danforth, (2006) [72]	Behaviours associated with ADHD	Presentation and symptoms		
	Aetiology including genetic, neurological and congenital factors – parents highly unlikely their parenting behaviour shaped ADHD	Aetiology		
	Environmental factors such as disorganised and loud environments, harsh parenting , harsh adult reactions and coercion escalate ADHD behaviour	Practical advice - behaviour	Practical advice - parenting techniques	
	Relevant technical terms: ABC, positive reinforcement, punishment, negative reinforcement, impact of immediate consequences	Practical advice - parenting techniques		
	Describe how many children with ADHD present with another separate childhood disorder called ODD	Co-morbidity - mental health (behaviour)		
	Characteristics of ADHD with examples from the therapists own experiences.	Presentation and symptoms		
	Executive functions may have little control over behaviour	Theories of cognition and ADHD		
	Influence of immediate rewards and poor inhibition.	Practical advice - cognition		
	High rate behaviour is resistant to extinction	Practical advice - behaviour		
Fogler et al., (2020)[76]	Diagnosis of ADHD	Diagnostic process		
	ADHD symptoms and impairments	Presentation and symptoms	Impact of ADHD	
	Genetic basis for ADHD	Aetiology		
	Evidence based treatment for ADHD	Treatment		
	Developmental course of ADHD	Developmental course of ADHD		
	Comorbidities	co-morbidity		
	ABC model of behaviour assessment	Practical advice - behaviour	Practical advice - parenting techniques	
	How ADHD impacts home – affects entire family system and impact of parental ADHD	Impact of ADHD	Social aspects	Impact of ADHD on the family
	Neurological basis of ADHD	Aetiology		
	ADHD medications	Treatment - medication		

	How to talk to children about having ADHD	Practical advice - communication skills		
	How ADHD affects children at school	Impact on Schooling and education		
	How medication can help children in school	Treatment - medication	Impact on Schooling and education	
	Strategies for building partnerships with teachers	Practical advice - education		
	How to design an individualised school plan using the ABC model	Practical advice - parenting techniques	Practical advice - education	
	Introduce parents to the daily report card	Practical advice - behaviour		
Foubister et al., (2020)[78]	Aetiology of ADHD	Aetiology		
	Presentation	Presentation and symptoms		
	Diagnosis	Diagnostic process		
	Evidence base for psychosocial interventions and medication	Treatment - medication	Treatment - psychosocial	
	Positive strategies to achieve and increase confidence in supporting children with ADHD	Practical advice - parenting techniques		
	Positive attention and building positive relationships, increase the chances of good behaviour recurring	Social aspects	Practical advice - behaviour	
	Managing challenging behaviour effectively, consider minor and more serious behaviours, what they communicate.	Practical advice - behaviour		
	ADHD reward program and planning for high risk outings and situations.	Practical advice - parenting techniques		
Garreta et al., (2018)[36]	Symptoms of adhd	Presentation and symptoms		
	Diagnostic criteria	Diagnostic process		
	Aetiology	Aetiology		
	Prevalence	Aetiology		
	Multimodal treatment	Treatment		
	Bibliography on ADHD	Practical advice - resources		
	Social learning, behavioural functioning and influencing factors			
	Techniques to increase positive behaviours, positive reinforcement, praise, chip economy	Practical advice - behaviour		
	Avoiding negative labels	Practical advice - sharing ADHD diagnosis with others		
	encourage and promote autonomy of the child with ADHD	Practical advice - behaviour		
	Extinction and ignoring negative behaviours	Practical advice - behaviour		
	Self-esteem, improve positive perception of the child with ADHD	Self-image/ self-esteem		
	Time out and repair	Practical advice - behaviour	Practical advice - relationships	
	Self-control of parents to children's misconduct	Practical advice - behaviour		
	Breathing based relaxation	Practical advice - mindfulness		
	Establishing limits	Practical advice - behaviour		
	Avoiding excessive or vague instructions	Practical advice - behaviour		
	Awareness of parenting cognitions and impact on parenting.	Practical advice - parent stress	Impact of ADHD	

	Communication, active listening and positive communication.	Practical advice - communication skills		
Gummus et al.,(2020) [79]	Causes of ADHD	Aetiology		
	Symptoms and signs of ADHD	Presentation and symptoms		
	Course and characteristics of the disorder according to age	Developmental course of ADHD		
	Treatment process	Treatment		
	Medications used in treatment and their side effects	Treatment - medication	Treatment - medication side effects	
	Correct and incorrect information about medications	Treatment - medication	Treatment - myths	
	Other symptoms that can co-exist about ADHD and their treatment and what parents can do about these issues	Co-morbidity	Practical advice - co-morbidity	
Haughan et al., (2022) [49] supportive measures in the school environment and homework				
	information about symptoms and causes of ADHD	Presentation and symptoms	Aetiology	
	how symptoms and associated problems change across different development stages	Developmental course of ADHD		
	treatment options, pharmacotherapy (including side effects)	Treatment - medication	Treatment - medication side effects	
	Supportive measures: planning and organising, supportive communication and use of helping aids	Practical advice - cognition	Practical advice - behaviour	
	Building a positive relationship between teacher and pupil :regular daily routines, use of a daily plan and week plans, clear communication/ short messages, use of rewards, learning by doing use of digital aids (calendars, alarms),	Practical advice - relationships	Practical advice - education	
	personal experiences from a parent,	Personal experience		
	local ADHD user-organization about rights that come with diagnosis.	Practical advice - resources		
Hantson et al., (2012)[28]	Identifying ADHD symptomatology	Presentation and symptoms		
	Pharmacological treatment	Treatment - medication		
	Importance of play and establishing positive relationship between parents and children	Practical advice - relationships		
	Praise and reward effectively	Practical advice - behaviour		
	Discipline and time out	Practical advice - parenting techniques		
	Importance of structure	Practical advice - behaviour		
	How to approach systems outside the homes, such as after school programs and other services	Practical advice - education	Practical advice - seeking help	
Kousha and Kakrodi (2019) [35]	Correcting parents' misconceptions about the nature of the disease	Myths of ADHD		
	Child-parent interactions	Practical advice - relationships		
	General principles of behavioural shaping	Practical advice - parenting techniques	Practical advice - behaviour	
	Token economy	Practical advice - parenting techniques		
	Managing misbehaviour	Practical advice - behaviour		
	Reduce child's inappropriate behaviour, planned ignoring, logical consequences	Practical advice - behaviour		
	Identifying children's problem solving	Practical advice - cognition		
	Behaviours in relation to the school	impact on schooling and education		
	Developing coping plans for high risk situations	Practical advice - behaviour		

	Symptoms of ADHD	Presentation and symptoms		
	Definition	Presentation and symptoms		
	Prevalence	Presentation and symptoms		
	Aetiology	Aetiology		
	General principles of treatment	Treatment		
Lindsrom et al., (2022) [40]	DSM definition, core symptoms and manifestations	Presentation and symptoms		
	Gender differences	Presentation in boys vs girls		
	Hypothesis of causes of ADHD	Aetiology		
	Information about diagnostic neuropsychiatric assessments	Diagnostic process		
	Treatment options	Treatment		
	Common maladaptive parent-child interaction patterns and their consequences	Practical advice - relationships	Practical advice - parenting techniques	Impact of ADHD on the family
	ADHD and peer relationships	Social aspects		
	The child's understanding of the disability	Self-image/ self-esteem		
	Situation of siblings	Impact of ADHD on the family		
	The social environment's support, or lack	Social aspects	Practical advice - resources	
	Lifestyle factors such as diet, physical activity, and screen time	Practical advice - lifestyle, stress, diet and exercise		
	Basic introduction to psychological and behavioural learning theories	Practical advice - parenting techniques		
	Behavioural parenting strategies to facilitate the management of the ADHD child's needs and behaviours	Practical advice - parenting techniques		
	Swedish laws regarding societal support services for families of children with ADHD	Practical advice - resources		
	Academic/ school based support	Practical advice - education		
	Financial support, municipal support and health care services.	Practical advice - resources		
Mikami et al., (2020) [38]	Understanding what ADHD is and how it impacts your child behaviours with peers	Presentation and symptoms	Social aspects	
	Identifying problem social behaviours	Social aspects	Practical advice - relationships	
	Gaining empathy for your child's social problems	Social aspects	Practical advice - gaining carer empathy	
	The most common conditions that co-occur with ADHD and how they are diagnosed	Co-morbidity		
	Understanding how your child's ADHD symptoms and social issues manifest over lifespan	Developmental course of ADHD	Social aspects	
	Academic challenges, reasons for these, and relation to social behaviour and peer's perceptions in school	impact on schooling and education	Social aspects	
	School rights and accommodations for ADHD, relation to peer problems	Practical advice - education	Practical advice - resources	
	Similarities and differences in how your child socially relates to different peers and adults, identifying patterns for child that facilitate good relationships	Social aspects	Practical advice - relationships	
	Medication and psychosocial treatment, and their effects on social behaviour	Treatment - medication	Treatment - psychosocial	
	Learning how to assess child's social problems and monitor treatment response	Social aspects	Practical advice - relationships	
	Recognizing the roles of multiple professions, how to facilitate communication between various professions	Practical advice – resources		

Miller and Brooker (2017) [98]	Primary and secondary symptoms of ADHD	Presentation and symptoms		
	Empirically supported treatments	Aetiology		
	Dealing with emotion dysregulation	Co-morbidity - mental health (emotions)		
	Managing am/pm routines	Practical advice - organisation		
	Giving effective commands	Practical advice - parenting techniques		
	Increasing tolerance for frustration	Practical advice - behaviour		
	Increasing positive behaviours	Practical advice - behaviour		
	Helping you child begin to be more aware and mindful	Practical advice - behaviour	Practical advice - mindfulness	
Monastra (2005) [100]	What is ADHD, symptoms	Presentation and symptoms		
	Causes of ADHD including role of genes	Aetiology		
	Rational for using medication	Treatment - medication		
	Strategies for reducing medication side effects,	Treatment - medication	Treatment - medication side effects	
	Tips for improving diet	Practical advice - lifestyle, stress, diet and exercise		
	Sleep and exercise habits	Practical advice - lifestyle, stress, diet and exercise		
	Importance of adequate support and accommodation at school	Practical advice - education		
	Practical strategies for addressing common behavioural, emotional and social problems	Practical advice - behaviour	Practical advice - parenting techniques	
Montoya et al.,(2014) [102]	Aetiology of ADHD	Aetiology		
	epidemiology	Presentation and symptoms		
	Symptomatology	Presentation and symptoms		
	Comorbidities	Co-morbidity		
	Diagnosis	Diagnostic process		
	Treatment	Treatment		
	Prognosis	Developmental course of ADHD		
	Reasons and sources of bad behaviour and influence on family interactions	Practical advice - behaviour		
	Situational circumstances	Practical advice - behaviour		
	Techniques orientated to improve behaviour	Practical advice - behaviour		
	Time optimisation	Practical advice - cognition	Practical advice - organisation	
	Reinforcements	Practical advice - parenting techniques	Practical advice - behaviour	
	Positive care	Practical advice - parenting techniques		
	Token system	Practical advice - behaviour		
	Techniques oriented to diminish inadequate behaviour	Practical advice - behaviour		
	Giving commands efficiently	Practical advice - parenting techniques		

	Outdoor time	Practical advice - lifestyle, stress, diet and exercise		
	How to handle inadequate behaviour in public places	Practical advice - behaviour	Practical advice - parenting techniques	
	Social skills	Social aspects		
	Emotional recognition and expression	Theories of cognition and ADHD		
	Social problem solving	Social aspects		
	Variable influencing attention problems	Theories of cognition and ADHD		
	Techniques and interventions to improve attention problems	Practical advice - cognition		
	How to support homework	Practical advice - education		
	Barley's decalog (rules are clear and brief, time represented externally, consequences delivered immediately, swiftly, greater magnitude, and more frequently, publically accountable, richer incentives required, rewards rotated frequently, use of anticipation time to rehearse rules and consequences, and interventions need continual monitoring and modification)	Practical advice - parenting techniques		
Risley et al.,(2020) [42]	Diagnosis of ADHD and underlying neurobiology, associated executive function deficits	Presentation and symptoms	Aetiology	Theories of cognition and ADHD
	Behavioural difficulties	Presentation and symptoms	Co-morbidity - mental health (behaviour)	
	Evidence based treatment options	Treatment		
	Principles of social learning theory and behaviour modification (including antecedents, behaviours consequences and their relationships, as well as schedules of reinforcement)	Practical advice - parenting techniques	Practical advice - behaviour	
	Use of descriptive praise, ignoring and special play time to manage ADHD behaviours	Practical advice - parenting techniques	Practical advice - behaviour	
Ryan et al., (2015)[108]	What is ADHD? Signs of ADHD, presentations of ADHD, differences between boys and girls, changes of symptoms over time.	Presentation and symptoms	Developmental course of ADHD	
	What is the route cause of ADHD	Aetiology		
	Talking to family and friends about ADHD, prejudices, and stereotypes	Practical advice - communication skills	Practical advice - sharing ADHD diagnosis with others	
	Looking after yourself	Practical advice - parent stress		
	Practical advice for home, routine, lists, visual timetables, focus on good behaviour, contracting, managing meltdowns, sleep, helping siblings	Practical advice - behaviour	Practical advice - parenting techniques	
	Helping your child's educational needs	Practical advice - education		
	Medication and behavioural therapies for ADHD	Treatment - medication	Treatment - psychosocial	
	Common myths	Myths of ADHD		
Shah et al.,(2019) [110]	What is ADHD?	Presentation and symptoms		
	Symptoms	Presentation and symptoms		
	What causes ADHD? Whose fault is it?	Aetiology	Practical advice - parent blame and guilt	
	Are two children with ADHD the same?	Presentation and symptoms		
	What other problems can a child with ADHD have?	Co-morbidity		
	Does ADHD improve with age?	Developmental course of ADHD		
	How is ADHD diagnosed?	Diagnostic process		

	Can ADHD be treated?	Treatment		
Shimabukuro et al.,(2017)[34]	nature and treatment of ADHD	Presentation and symptoms	Treatment	
	history of ADHD	Historical aspects		
	presumed causes	Aetiology		
	core symptoms	Presentation and symptoms		
	associated difficulties	co-morbidity		
	adolescent and adult outcomes and prognosis	Developmental course of ADHD	Impact of ADHD	
	treatment options for ADHD	Treatment		
	details on where to obtain further information about ADHD,	Practical advice - resources		
	handouts to help them inform others about ADHD. correct any parental misperceptions about the disorder, help parents generate realistic expectations of their child, and to empower them in discussing their child's difficulties with others.	Practical advice - sharing ADHD diagnosis with others	Myths of ADHD	
Svanborg, Thernlund, Gustafsson, Hägglöf, Schnacht, et al., (2009)[113]	Knowledge and understanding of ADHD and implications on the child’s everyday functioning	Presentation and symptoms	Impact of ADHD	
	Awareness of how caregiver can adjust the environment to the child’s functional level	Practical advice - parenting techniques		
	Guidance on how to facilitate and promote positive parent-child interaction	Practical advice - parenting techniques	Practical advice - relationships	
	Positive behavioural patterns	Practical advice - behaviour		
	Behaviour modification principles in handling problem behaviour	Practical advice - behaviour	Practical advice - parenting techniques	
	Available social and educational support	Practical advice - resources	Practical advice - education	
Treacy et al.,(2005)[111]	Nature of stress and how to recognise it, stresses associated with parenting a child with ADHD, effects of stress on parents and parenting practices, effective and ineffective ways of managing stress	Impact of ADHD on the family	Practical advice - parenting techniques	Practical advice - parent stress
	History of ADHD,	Historical aspects		
	presumed causes,	Aetiology		
	core symptoms and associate difficulties,	Presentation and symptoms	Impact of ADHD	
	adolescent and adult outcomes and prognosis,	Developmental course of ADHD		
	treatment options,	Treatment		
	correct parental misperceptions, generate realistic expectations of their child, empower them in discussing child’s difficulties with others	Myths of ADHD	Practical advice - parenting techniques	Practical advice - sharing ADHD diagnosis with others
	Family educational and financial entitlements and available community resources	Practical advice - resources		
	Problem solving skills, problem identification, problem specification, solution generation, evaluation of alternative solutions, implementation of chose solutions, and outcome evaluation	Practical advice - cognition	Practical advice - behaviour	Practical advice - parenting techniques
	Common cognitive errors, impact on emotions and behaviour, identifying own faulty cognitions and replacing them with more adaptive thoughts.	Theories of cognition and ADHD	Co-morbidity - mental health	Practical advice - cognition
	Giving effective commands to children with ADHD, enhancing communication between partners, communicating effectively with school and health professionals	Practical advice - parenting techniques	Practical advice - seeking help	
	Self care skills, time management, relaxation techniques	Practical advice - parent stress	Practical advice - organisation	
	Appropriate use of discipline,	Practical advice - parenting techniques		

Van den Hoofdakker, et al., (2007)[85]	ADHD and behaviour management principles	Practical advice - behaviour	Practical advice - parenting techniques	
	Influence of the child with ADHD on family, marital relationship, brothers and sisters, the neighbourhood	Social aspects	Impact of ADHD on the family	
	Distinguishing parental cognitions and emotions	Practical advice - parent stress		
	Antecedent-behaviour-consequence – functional analysis of behaviour	Practical advice - behaviour		
	observational skills	Practical advice - parenting techniques		
	Understanding factors that may interfere with behavioural change (such as dysfunctional parental cognitions, parenting stress, other stressors in the family).	Practical advice - parent stress		
	Structuring the environment	Practical advice - parenting techniques		
	Reinforcing positive behaviours through attention and praise	Practical advice - parenting techniques		
	Setting rules	Practical advice - parenting techniques		
	Giving commands, anticipating misbehaviours	Practical advice - behaviour		
	Increasing children's self-esteem	Self-image/ self-esteem		
	Ignoring Misbehaviours	Practical advice - behaviour		
	Employing punishment	Practical advice - parenting techniques		
	Reward-orientated token system	Practical advice - parenting techniques		
	Anticipating future problems.	Practical advice - behaviour		
Zheng et al., (2020)[115]	Knowledge about ADHD, aetiology, manifestation, harm and treatments	Presentation and symptoms	Aetiology	Impact of ADHD
	Medication: side effects and how to manage	Treatment - medication		
	teaching parents how to use SNAP-IV	Practical advice - behaviour		
	teaching parents behavioural strategies to manage conduct problems	Practical advice - co-morbidity		
	positive and negative reinforcement,	Practical advice - parenting techniques		
	teaching parents to combine procedure and behaviour management techniques,	Practical advice - parenting techniques		
	to help attention,	Practical advice - cognition		
	impulse control,	Practical advice - behaviour		
	self regulation and working memory	Practical advice - cognition		

Table 4: Parents/carers and children with ADHD

Study	Psychoeducation descriptions	Code 1	Code 2	Code 3
Dopfner et al.,(2004)[73]	Delivered to child, family and teacher:			
	Individual causes and maintaining conditions: genetic factors, problems of the parents or parental reactions to the problem behaviour and reactions of teachers to the child	Aetiology	Impact of ADHD	Practical advice - parenting techniques
	Stressors for the family, parents and child, including partner problems and emotional problems	Impact of ADHD on the family	Practical advice - relationships	
Ferrin et al., (2014)[74]	What is ADHD	Presentation and symptoms		
	Core symptoms and diagnostic procedures	Presentation and symptoms	Diagnostic process	
	Aetiology, maintaining and perpetuating factors	Aetiology		
	Comorbidities in ADHD	co-morbidity		
	Prognosis and outcome, ADHD in adolescents and adults	Developmental course of ADHD	Impact of ADHD	
	Pharmacology treatments, stimulants and non-stimulants	Treatment - medication		
	Non-pharmacological treatments: diets, supplements, cognitive and behavioural treatments	Treatment - psychosocial	Treatment	
	Dealing with everyday life problems at home	Practical advice		
	Dealing with everyday life problems at school	Practical advice - education		
Ferrin et al., (2020)[46]	Symptoms of ADHD	Presentation and symptoms		
	What is ADHD and what is not?	Presentation and symptoms		
	Diagnosis	Diagnostic process		
	Subtypes of ADHD	Presentation and symptoms		
	Aetiology, genes, environmental factors	Aetiology		
	Myths, misconceptions and stereotypes	Myths of ADHD		
	Co-morbidities of ADHD, depression, anxiety, ODD, CD, substance abuse, tic disorders and learning disabilities	co-morbidity - mental health (emotions)	Co-morbidity - substance use	co-morbidity - mental health (behaviour)
	Prognosis, development through the lifespan	Developmental course of ADHD	Impact of ADHD	
	Pharmacological treatment	Treatment - medication		
	Non-pharmacological treatments	Treatment - psychosocial		
	Basic parenting techniques	Practical advice - parenting techniques		
	Managing the difficult child	Practical advice - behaviour		
	Helping the ADHD child at school and with homework	Practical advice - education		
Fields and Hale (2011)[75]	What is ADHD?	Presentation and symptoms	Aetiology	
	Communication skills	Practical advice - communication skills		
	Behavioural management	Practical advice - behaviour		
	Problem solving	Practical advice - behaviour		
	Family anger management	Practical advice - relationships		
Haugan et al., (2022)[49]	typical symptoms	Presentation and symptoms		
	causes	Aetiology		
	advice on family communication,	Practical advice - communication skills		
	daily structure,	Practical advice - behaviour		
	diet	Practical advice - lifestyle, stress, diet and exercise		
	relevant pharmacological treatment options	Treatment - medication		

Hogue et al., (2021)[81]	CASH-AA Psychoeducation			
	prevalence rates,	Aetiology		
	behavioural symptoms,	Presentation and symptoms		
	common impacts on developmental functioning,	Developmental course of ADHD		
	anti-stigma message while encouraging the teen to take ownership of ADHD-related characteristics,	Stigma and prejudices	Practical advice - behaviour	
	neurobiology of ADHD,	Aetiology		
	potential benefits of medication	Treatment - medication		
	Define components of executive functioning including working memory, behavioural inhibition, emotional control, planning and organisation, and analytic skills	Theories of cognition and ADHD		
	Influences on academic achievement: intelligence, EF skills, ADHD behavioural symptoms.	impact on schooling and education		
	Prevalence rates for learning disabilities	co-morbidity - learning disability		
	MIP protocol[82] also has:			
	ADHD medication facts: common benefits, course and side effects of medications, details the trial and error approach to appropriate dosing, summarizes key factors that inform decision-making about medication initiation.	Treatment - medication	Treatment - medication side effects	
	Positive and negative personality and social characteristics associated with ADHD	Social aspects	Strengths and positive elements of ADHD	
Lantz et al., (2021) [45]	Aetiology and neurobiology basis	Aetiology		
	Potential difficulties and strengths related to ADHD	Impact of ADHD	Strengths and positive elements of ADHD	
	Emotions	Theories of emotions		
	Strategies to deal with everyday life and ADHD symptoms: stop and think, get organised, use tools, and use support and coaching	Practical advice - parenting techniques		
	Importance of sleep, food and physical activity	Practical advice - lifestyle, stress, diet and exercise		
Lopez et al., (2005)[54]	Diagnosis given and how it was made	Diagnostic process		
	Information on treatment options: pharmacological, psychotherapeutic and other psychosocial options	Treatment - medication	Treatment - psychosocial	
	Medication information benefits and disadvantages of specific medication options, proper administration of the medication, how long for benefits to be seen.	Treatment - medication	Treatment - adherence	
	Importance of asking the physician questions and communicating openly, questions they might ask	Practical advice - seeking help		
	Behavioural strategies for effective management of behaviour	Practical advice - parenting techniques		
	Changing the environment and behaviour, working with the school, and teaching and encouraging social skills.	Practical advice - education	Practical advice - seeking help	Practical advice - communication skills
	Communication skills, mental health and substance use,	Practical advice - communication skills	Co-morbidity - substance use	co-morbidity - mental health
	stigma	Stigma and prejudices		
	rewards for children and adolescents	Practical advice - behaviour		
	Encourage questions and develop questions for their psychiatrist	Practical advice - seeking help		
	advice for improving organisational skills, time management and completion of school assignments	Practical advice - cognition	Practical advice - behaviour	Practical advice - education
	Printed handouts to parents: signs and symptoms of the disorders, aetiology, and prognosis	Presentation and symptoms	Aetiology	Developmental course of ADHD

	References to additional sources of information and support	Practical advice - resources		
	Types of medications used to treat the disorder, potential benefits of the medication, common side effects of the medications, the time frame for medication to work, a general description of the pharmacological mechanisms, what the physician may do if the child/ adolescent foes not improve or has unacceptable side effects	Treatment - medication	Treatment - medication side effects	
McCarty et al., (2015)[93]	Review of assigned pre-study readings on the Diagnosis and phenomenology of ADHD	Presentation and symptoms	Diagnostic process	
	Common myths about ADHD	Myths of ADHD		
	Management: a multi modal approach	Treatment		
	Review of medication choices	Treatment - medication		
	Medication side effects	Treatment - medication side effects		
	Anatomical areas of the CNS implicated in the expression of ADHD	Aetiology		
	Neurochemical transmission in the CNS implicated in the actions of ADHD medications	Aetiology	Treatment - medication	
	The prefrontal cortex and human behaviour	Theories of cognition and ADHD		
	Executive functions in ADHD and learning	Theories of cognition and ADHD	impact on schooling and education	
	The prefrontal cortex executive functions, and ADHD: selective attention, sustained attention, hyperactivty, impulsivity	Theories of cognition and ADHD		
	Types and prevalences of comorbidities	Co-morbidity		
	Adjunctive problems in ADHD: motor development, fine motor skills, body boundaries, social awareness	co-morbidity	impact of ADHD	
	Core symptoms of ADHD: tip of the iceberg	Developmental course of ADHD		
	Impact of ADHD on development; inattention and hyperactivity over time	Developmental course of ADHD		
	Other consequences during development	Developmental course of ADHD		
	ADHD and adolescence	Developmental course of ADHD		
Nagae et al., (2019) [103]	What is adherence?	Treatment - medication	Treatment - adherence	
	Let me think about my medication: to think about what the positive and negative aspects of taking the medication are for you. To learn about the differences between medications	Treatment - medication		
	Let me learn about ways of managing my medication: to understand how your medication is usually managed. To try taking over part of your medication management yourself.	Treatment - medication		
	Mangaging my medication myself: find ways to avoid forgetting to take your medication	Treatment - adherence		
Schoenfelder et al., (2020) [44]	Problems associated with ADHD	Impact of ADHD		
	Causes, aetiology	Aetiology		
	Evidenced based treatments	Treatment - evidence based medicine		
	Normative adolescent psychosocial development	neurotypical adolescent development		
	Health risk behaviours associated with ADHD (e.g., substance abuse, risky driving, sexual behaviour, overeating).	Co-morbidity - substance use	Co-morbidity - lifestyle, stress, diet and exercise	

Table 5: Children with ADHD

Author	Psychoeducation descriptions	code 1	code 2	code 3
Meyer, Ramklint, et al.,(2022)[90]	What is ADHD?	Presentation and symptoms		
	Aetiology,	Aetiology		
	neurobiology,	Aetiology		
	symptomatology,	Presentation and symptoms		
	difficulties and strengths with ADHD	Impact of ADHD	Strengths and positive elements of ADHD	
	Normalising symptoms using famous people impact on)	Strengths and positive elements of ADHD	Self-image/ self-esteem	
	Take charge over your daily life: how to structure daily life routines and the importance of sleep, food and activity, stress management and problem solving skills.	Practical advice - behaviour	Practical advice - lifestyle, stress, diet and exercise	Practical advice - cognition
	Take charge over your ADHD: using the problem solving model, following the structure: stop and think, get organized, use tools, use support and coaching.	Practical advice –cognition		
MacKay and Corkum(2006)[43]	Core ADHD symptoms and associated problems	Presentation and symptoms	Co-morbidity	
	What is ADHD	Aetiology	Presentation and symptoms	
	Brain structures that have been implicated in ADHD	Aetiology		
	Every brain is "unique" (highlighting potential strengths and challenges)	Impact of ADHD	Strengths and positive elements of ADHD	
	Myths about the aetiology of ADHD	Myths of ADHD		
	Use of medication in treatment of ADHD	Treatment - medication		
	How do you relax?	Practical advice - lifestyle, stress, diet and exercise		
	Who can help with ADHD?	Practical advice - seeking help		
	Video which presents ADHD from a child's point of view	Personal experience		
Hantson et al., (2012)[28]	Introducing oneself			
	Joining in	Practical advice - behaviour		
	Responding to teasing	Practical advice - communication skills		
	Staying out of fights	Practical advice - behaviour		
	Identifying teasing and bullying	Social aspects		
	Walking away from a negative situation	Practical advice - behaviour		
	I feel statements	Practical advice - behaviour		
	Stop and count to 10	Practical advice - behaviour		
	Body language	Practical advice - behaviour	Social aspects	
	Evaluating consequences	Practical advice - behaviour		

Table 6: Adults with ADHD

Author	Psychoeducation descriptions	Code 1	Code 2	Code 3
Anastopoulos and King (2015)[47]	What is ADHD?	Aetiology	Presentation and symptoms	
	What causes ADHD?	Aetiology		
	Assessment of ADHD symptoms	Diagnostic process		
	How does ADHD affect school? Does ADHD only affect school?	impact on schooling and education		
	Depression, anxiety and other things that may go with ADHD	Co-morbidity - mental health	co-morbidity - mental health (emotions)	
	Sex, drugs and ADHD	Co-morbidity - substance use		
	What medications are used to treat ADHD?	Treatment - medication		
	Is medication the only way to treat ADHD?	Treatment - psychosocial		
	A look into the future?	Developmental course of ADHD		
Bachmann et al., (2018)[64]	Symptoms of ADHD	Presentation and symptoms		
	Causes of ADHD	Aetiology		
	Treatments of ADHD	Treatment		
	Potentials and problems of people with ADHD in social life	Social aspects		
	How do I deal with me? Self image and self esteem	Self-image/ self-esteem		
	Chaos and control – self organisation in everyday life	Practical advice		
	Stress management – basics of stress and prevention	Practical advice - lifestyle, stress, diet and exercise		
	Mood regulation and impulse control	Co-morbidity - mental health (emotions)		
	Self modification of problematic behaviour	Practical advice - behaviour		
	ADHD diagnosis.	Diagnostic process		
Bjork et al., (2020)[41]	Physical exercise recommendations	Practical advice - lifestyle, stress, diet and exercise		
	Diet recommendations	Practical advice - lifestyle, stress, diet and exercise		
	Physical health and how to handle and prevent symptoms of physical illness	Co-morbidity - physical health		
	Mental health and how to handle and prevent symptoms of mental illness	Co-morbidity - mental health		
	Cognitive impairments and how to cope with them	Practical advice - cognition	Theories of cognition and ADHD	
	Lifestyle disorders and how to prevent them (sleep problems, stress. Metabolic syndrome)	Co-morbidity - physical health	Practical advice - physical health	
	Working life and sick leave, financial support	Work and finances		
	Social relationships	Social aspects		
	Risky living habits such as use of alcohol/ drugs and smoking	Co-morbidity - substance use		
Hartung et al., (2022)[32]	Evidence based assessment of ADHD symptoms	Diagnostic process	Presentation and symptoms	
	Core symptoms of ADHD	Presentation and symptoms		
	History	Historical aspects		
	Aetiology	Aetiology		
	Development course of the disorder	Developmental course of ADHD		
	Deficits in executive functioning may affect college students	Theories of cognition and ADHD	impact on schooling and education	
	Things that exacerbate symptoms of inattention and hyperactivity/impulsivity	Practical advice – behaviour		
	Psychosocial and medication treatments for ADHD	Treatment – medication	Treatment - psychosocial	
	Reviewed evidence based approaches	Treatment - evidence based medicine		
	Discussion of participants experience with treatment, and encouragement to consult with their medical providers if unhappy	Treatment - medication	Practical advice - seeking help	
	Discourage participants from sharing their medication and provided with examples of both physical health and legal consequences	Stimulant diversion		
	Symptoms of ADHD	Presentation and symptoms		

De Oliveira et al., (2018)[104]	Impact of ADHD on college students	impact on schooling and education		
	Causes of ADHD	Aetiology		
	Neurobiology of ADHD	aetiology		
	How ADHD is diagnosed	Diagnostic process		
	Myths of ADHD	Myths of ADHD		
	Study strategies for ADHD	Practical advice - education		
	Medical and non medical treatments for ADHD	Treatment - medication	Treatment - psychosocial	
Vidal et al., (2013)[31]	Myths and realities in ADHD	Myths of ADHD		
	Diagnosis and characteristics of ADHD	Presentation and symptoms	Diagnostic process	
	ADHD causes and treatments	Aetiology	Treatment	
	Implication of a family member	Impact of ADHD on the family		
	Positive and negative symptoms	Strengths and positive elements of ADHD		
	Relaxation in ADHD	Practical advice - lifestyle, stress, diet and exercise		
	Cognitive model of ADHD	Theories of cognition and ADHD		
	Behavioral factors	Presentation and symptoms		
Hirvikoski et al., (2015)[27]	Understanding of the ADHD diagnosis, common difficulties, comorbidity and strengths	Diagnostic process	Presentation and symptoms	co-morbidity
	Pharmacology and psychological treatment	Treatment - medication	Treatment - psychosocial	
	Lifestyle factors sleep, stress diet and exercise/ Connection between general lifestyle factors and ADHD symptom severity	Practical advice - lifestyle, stress, diet and exercise		
	Strategies and cognitive aids to ease life of individuals with executive difficulties	Practical advice - cognition		
	Living with ADHD, acceptance and lived experience	Practical advice	Personal experience	
	ADHD symptoms impact on social behaviours and relationships, positive and negative aspects of ADHD in relationships	Social aspects	Strengths and positive elements of ADHD	
	ADHD at work, support measures provided by employment services and how the workplace may be adjusted based on ADHD symptoms	Work and finances		
	Various support measures society may provide for individuals with ADHD	practical advice - resources		
Hoxhaj et al., (2018) [39]	ADHD (symptoms, causes and treatments)	Presentation and symptoms	Aetiology	Treatment
	Potentials and problems of people with ADHD in social life	Social aspects		
	How do I deal with me? Self-image and self-esteem	Self-image/ self-esteem		
	Chaos and control – self organization in everyday life	Practical advice		
	Stress management – basics on stress and stress prevention	Practical advice - lifestyle, stress, diet and exercise		
	Mood regulation and impulse control	Co-morbidity - mental health (emotions)		
	Self modification of problematic behaviour	Practical advice - behaviour		
In de Braek et al., (2017)[87]	What is ADHD? What does ADHD mean for them [the participants]?	Presentation and symptoms		
	Attention	Theories of cognition and ADHD		
	Automatic versus nonautomatic tasks	Theories of cognition and ADHD		
	Memory functioning	Theories of cognition and ADHD		
	Own goals	Practical advice - cognition		
	Prioritizing	Practical advice - cognition		
	Planning	Practical advice - cognition		
	Making a week plan/ terms for an adequate plan	Practical advice		
	Doing one thing at once/ adapting structure	Practical advice		
	Work/ relationships/ finances	Work and finances		
Jans et al., (2015)[37]	Neurobiology of ADHD	Aetiology		
	Links to the patient's symptoms in everyday life	Presentation and symptoms		

	Recommendations on useful websites and books on ADHD	Practical advice - resources		
	Mindfulness and meditative exercises to teach mindfulness skills.	Practical advice - mindfulness		
	Linking mindfulness skills to the control of ADHD	Practical advice - mindfulness		
	Disorganised behaviours and devolving organisational strategies	Practical advice - cognition		
	Planning how to implement these strategies to daily life	Practical advice - behaviour		
	Theory of emotions; links between cognition and emotion	Theories of emotions		
	Education on depressive symptoms and treatment	Co-morbidity - mental health (emotions)	Treatment - mental health	
	Pharmacotherapy of ADHD	Treatment - medication		
	Aspects of impulsivity, how to use mindfulness and functional analysis skills to improve impulse control, thinking about consequences of behaviour	Practical advice- behaviour	Practical advice - mindfulness	
	Theoretical model for stress and stress reactions, reflecting on individual dysfunctional and functional stress management in the past	Practical advice - lifestyle, stress, diet and exercise		
	Symptoms of substance abuse	Co-morbidity - substance use		
	personal risk behaviours (internet use, exercise, sensation seeking activities, sexuality, overeating included), focus on change when needed	Practical advice - lifestyle, stress, diet and exercise		
	Impact of ADHD on the patient's personal biography and self respect, individual strategies to improve relationships	Self-image/ self-esteem	Social aspects	
	How to inform others on the diagnosis of adhd and implications for the patient	Practical advice - sharing ADHD diagnosis with others		
Pettersson et al., (2017)[106]	ADHD and restlessness/ hyperactivity	Presentation and symptoms		
	Rational for goal planning	Practical advice - cognition		
	ADHD and impulsivity	Presentation and symptoms		
	Rational for behavioural analysis and mindfulness	Treatment – psychosocial	Practical advice - mindfulness	
	Perception of time.	Theories of cognition and ADHD		
	Rationale for time management and mindfulness	Treatment – psychosocial		
	Attention, working memory and distraction	Theories of cognition and ADHD		
	Rationale for gauging attention span and reducing distractors.	Practical advice – cognition		
	Rational for organisation and planning	Practical advice – cognition		
	Procrastination and passivity	Practical advice – behaviour		
	Rationale for problem solving and behaviour activation	Practical advice – behaviour		
	Regulation of emotions	Co-morbidity - mental health (emotions)		
	Rational for acceptance and mindfulness	Practical advice - mindfulness		
	The cognitive model. Theory of automatic thoughts. "thought traps". The relationship between thoughts emotions, physiological reactions, and behaviour.	Theories of cognition and ADHD		
	Aggression and acting out.	Co-morbidity - mental health (behaviour)		
	Rational for anger control training.	Practical advice - behaviour		

Table 7: Teachers

Author	Psychoeducation descriptions	Code 1	Code 2	Code 3
Aguiar et al., (2014)[62]	Clinical vignettes on frequent misconceptions	Myths of ADHD		
	ADHD symptoms presentation at school and aetiology	Presentation and symptoms	Aetiology	impact on schooling and education
	Strategies for managing ADHD in school	Practical advice - education		
Corkum et al., (2019)[69]	Common myths about ADHD	Myths of ADHD		
	Impact, aetiology and effective treatments	Impact of ADHD	Aetiology	Treatment
	Teachers role in working with students with ADHD	Practical advice - education		
	Importance of home-school cooperation using a team approach	Practical advice - education	Practical advice - communication skills	
	Structuring physical classrooms	Practical advice - education		
De Jongh et al., (2019)[89]	defining ADHD	Presentation and symptoms		
	the features, prevalence and aetiology	Presentation and symptoms	Aetiology	
	symptoms and risk factors associated with ADHD	Aetiology	Presentation and symptoms	
	the co-morbid conditions related to ADHD	co-morbidity		
	risk factors aggravating the condition: dietary factors, nutritional deficiencies, biological factors, abnormal lighting exposure to environmental toxins and psychological factors	Aetiology	Co-morbidity - lifestyle, stress, diet and exercise	
	identification and diagnosis of ADHD	Diagnostic process		
	coping strategies for teachers managing ADHD	Practical advice - education		
	multimodal treatment of ADHD including pharmacological and non-pharmacological interventions, dietary modifications, psychosocial education, behavioural modification, adapt the school environment	Treatment - medication	Treatment - psychosocial	Practical advice - education
	The emergent literacy topics comprised the impact of ADHD on emergent literacy and reducing the risk of educational complications such as emergent literacy.	Impact of ADHD	impact on schooling and education	
Haugan et al., (2022)[49]	Teacher and parent: supportive measures in the school environment and homework	Practical advice - education		
	information about symptoms and causes of ADHD	Presentation and symptoms	Aetiology	
	how symptoms and associated problems change across different development stages	developmental course of ADHD		
	treatment options, pharmacotherapy (including side effects)	Treatment	Treatment - medication	Treatment - medication side effects
	Supportive measures: planning and organising, supportive communication and use of helping aids	Practical advice - cognition	Practical advice - communication skills	
	Building a positive relationship between teacher and pupil :regular daily routines, use of a daily plan and week plans, clear communication/ short messages, use of rewards, learning by doing use of digital aids (calendars, alarms),	Practical advice - relationships	Practical advice - communication skills	Practical advice - education
	personal experiences from a parent,	Personal experience		
	local rights that come with diagnosis.	Practical advice - resources		
Jones and Chronis-Tuscano (2008)[88]	General overview of ADHD including identification and diagnosis	Presentation and symptoms	Diagnostic process	
	Evidence based treatment for ADHD (including pharmacological and non pharmacological)	Treatment - medication	Treatment - psychosocial	

	Classroom behaviour management strategies	Practical advice - behaviour	Practical advice - education	
Lasisi et al., (2017)[91]	Symptoms of ADHD	Presentation and symptoms		
	Associated impairment	Impact of ADHD		
	Other conditions that need to be excluded	Diagnostic process		
	Behavioural interventions	Practical advice - behaviour		
	Medication	Treatment - medication		
	Classroom management strategies	Practical advice - education		
Latouche and Gascoigne, (2019)[92]	ADHD aetiology	Aetiology		
	Neuropsychological and executive functioning impairments	Theories of cognition and ADHD		
	Assessment of ADHD	Diagnostic process		
	Symptoms of ADHD	Presentation and symptoms		
	diagnosis of ADHD	Diagnostic process		
	Course/ consequences	developmental course of ADHD	Impact of ADHD	
	Cultural conceptualizations	Stigma and prejudices		
	Treatment	Treatment		
Lopez et al., (2005)[54]	Common educational and behavioural difficulties that result from the disorder	impact on schooling and education		
	Concrete suggestions for ways that the school can facilitate success at school	Practical advice - education		
Miranda et al., (2006)[48]	Nature of ADHD	Presentation and symptoms		
	Incidence of ADHD	Presentation and symptoms		
	Effects of ADHD on learning	Theories of cognition and ADHD		
	Basic features of the disorder regarding impulse control, activity and attention	Theories of cognition and ADHD	Presentation and symptoms	
	Common problems associated with hyperactivity	co-morbidity		
	Early identification, the developmental progression and long term prognosis	developmental course of ADHD	Impact of ADHD	Diagnostic process
	Educational demands of hyperactive students	impact on schooling and education		
	Behaviour modification procedures to increase desirable behaviours: positive reinforcement, the Premack principle and token systems	Practical advice - behaviour		
	Teaching techniques designed to decrease inappropriate behaviours: extinction, time-out and response cost	Practical advice - education	Practical advice - behaviour	
	Guidelines on instructional management procedures for students with ADHD	Practical advice - education	Practical advice - behaviour	
	Patterns to arrange the physical space	Practical advice		
	The presentation of explanations	Practical advice - communication skills		
	The directions and feedback in the performance of tasks and examinations	Practical advice - communication skills		
	Recognition and definition of ADHD	Presentation and symptoms		
Sarraf et al., (2011)[109]	Epidemiology	Presentation and symptoms		
	Aetiology	Aetiology		
	Differential diagnosis	Diagnostic process		

	Accompanied disorders	co-morbidity		
	Prognosis	developmental course of ADHD	Impact of ADHD	
	Treatment	Treatment		
	Unreal beliefs about the disorder	Myths of ADHD		
	ADHD impulse and hyperactivity control	Practical advice - behaviour		
	Strategies for attention increase in classrooms and schools	Practical advice - education	Practical advice - cognition	
	The manner of teacher-parent and teacher-psychiatrist relationship.	Practical advice - communication skills	practical advice - seeking help	
Zheng et al., (2020)[115]	knowledge of ADHD,	Presentation and symptoms	Aetiology	
	behavioural strategies, classroom behaviour management,	Practical advice - education	Practical advice - behaviour	
	teaching teachers how to use scaffolding to promote development of self regulation	Practical advice - education	Practical advice - behaviour	
